# Transcriptional landscape of metal-resistant *Cupriavidus metallidurans*: one sigma factor to rule them all

**DOI:** 10.1128/jb.00219-26

**Published:** 2026-07-27

**Authors:** Dietrich H. Nies, Jan Grau, Grit Schleuder, Cornelia Große

**Affiliations:** 1 Molecular Microbiology, Martin-Luther-University Halle-Wittenberg, Halle, Germany; 2 Computer Science, Martin-Luther-University Halle-Wittenberg, Halle, Germany; National Institutes of Health, Bethesda, Maryland, USA

**Keywords:** promoter, zinc, sigma factor

## Abstract

*Cupriavidus metallidurans* is able to grow in the presence of transition metal mixtures with high concentrations and varying compositions. This ability is mediated by a multitude of metal transport and repository systems. To reveal the genetic regulation underlying these physiological processes, 12,914 transcriptional start sites (TSSs) in metal-stressed and metal-starved cells were determined, and regulatory tags assigned to each TSS. Subsequently, the sense and antisense transcripts downstream of these TSSs were associated with the respective TSS. In this way, the impact of each TSS on genes via sense and antisense transcripts was derived, and a comprehensive transcriptional landscape of *C. metallidurans* was created. Depending on the replicon, 42%–65% of the TSSs in *C. metallidurans* were regulated under conditions of changing metal availability, which resulted in significant reorganization of the transcriptome in the bacterium. Promoter consensus motifs for −10 and −35 sites were identified in a pattern search in the upstream region of the TSSs. The results indicate that 83.6% of the TSSs in *C. metallidurans* have promoters that are mainly used by the housekeeping sigma factor RpoD. Surprisingly, the majority of these promoters are sba (sliding-blocking-another sigma factor) promoters with a correctly positioned −10 site but a −35 site up- or down-stream of the optimal −35 position. The data indicate that RpoD-dependent RNA polymerases dominate transcription initiation in *C. metallidurans,* interacting at sba promoters in a complex pattern with additional promoter elements, regulatory proteins, and, in some cases, other sigma factors, which is a previously unknown regulatory mechanism.

**IMPORTANCE:**

Textbook knowledge suggests that RpoD and related sigma factors use −35 and −10 motifs upstream of a TSS to bind to the promoter, melt the DNA, and initiate RNA synthesis, with different sigma factors recognizing different motifs. This is not the case in *Cupriavidus metallidurans*. The majority of its promoters have a correctly positioned −10 site but an incorrectly positioned −35 site. At these “sba promoters,” other factors are needed for the correct positioning of the RpoD-bound RNA polymerase (RNAP) at the −10 site. In some cases, alternative sigma factors compete with RpoD-RNAP for promoter-binding. The complete transcriptional landscape revealed in *C. metallidurans* an altogether novel and complicated regulatory interaction at sba promoters.

## INTRODUCTION

*Cupriavidus metallidurans* strain CH34 serves as a model system for our understanding of homeostasis of multiple transition metals in bacteria (1–4). This beta-proteobacterium occurs in metal-rich environments, such as auriferous soils or zinc deserts (5–8). Nevertheless, *C. metallidurans* is also able to survive under metal-starvation conditions.

As outlined elsewhere (3, 9), the homeostasis mechanisms of Zn(II) and other transition metal cations rely on a flow-equilibrium of metal uptake and efflux reactions (10, 11), which competes with the metal-binding capacity of the cytoplasm, through glutathione, polyphosphate, the overall metal-binding capacity of the proteome, and the zinc repository (10, 12). For example, under starvation conditions, the Zur regulon components ensure that zinc is more efficiently used and in part substituted by cobalt ions (13–16), while at higher transition-metal concentrations, inner membrane and transenvelope efflux systems become activated (17–20), for instance, the cobalt-zinc-cadmium export systems CzcCBA or for cobalt-nickel CnrCBA (3).

The genes encoding the systems governing metal homeostasis in *C. metallidurans* are mostly located on replicons or genomic islands acquired by horizontal gene transfer (2, 21–24). Besides its chromosome, the bacterium also harbors a chromid and two plasmids, pMOL30 and pMOL28 (4). Genomic (21, 25), global transcriptomic (26, 27), and proteomic studies (28) have revealed how *C. metallidurans* adapts to different concentrations of transition-metal cations, which are all toxic at higher concentrations. Group 4 sigma factors of the extracytoplasmic function family (ECF) of this bacterium are also involved in global metal homeostasis (29–33).

Sigma factors are at the top level of hierarchically organized regulatory processes in bacterial cells because they are essential for transcription initiation (34–36). All bacteria possess at least the housekeeping sigma factor, RpoD (alternative names SigA or sigma-70), which contains several conserved domains that are enumerated from the N- to the C-terminus of the polypeptides. Most important are the domains 2, 3, and 4, which are present in most RpoD-related sigma factors (35–37). For transcription initiation to occur, RpoD must bind the RNA polymerase (RNAP) core enzyme to form the RpoD-dependent and transcription-initiation-competent RNAP holoenzyme. Subsequently, RpoD-RNAP binds to the promoter region of the DNA to form the closed binary complex. RpoD domain 2 recognizes the −10 element of the promoter sequence with the consensus sequence TATAAT, rotates the second A and last T out of the DNA double strand, binding both bases into well-defined pockets of the sigma factor (38). This leads to an efficient melting of the DNA, the formation of the open binary complex between promoter DNA and RpoD-RNAP, with subsequent transcription initiation (34).

Up to five other components of the RpoD-dependent promoters determine the specific interaction between domain 2 of RpoD-RNAP and the −10 site. The three main components are: (i) the −35 element with the consensus sequence TTGACA that interacts with domain 4; (ii) the extended −10 site TGN directly upstream of the −10 motif, which interacts with domain 3; and (iii) the first part of the discriminator GGGA directly downstream of the −10 site interacting with domain 1.2 (39–41). Finally, (iv) the AT-rich UP element upstream of the −35 site, which interacts with the carboxy-terminal domains of the alpha subunit of the RNAP, can influence the positioning of the holoenzyme at the −10 site; and even (v) DNA-binding activators, which interact with the RNAP (42), can impact the interaction. For RpoD-dependent promoters, one of these positioning factors may be sufficient, but several can combine to increase the efficiency of transcription initiation (34).

Most bacteria have “alternative sigma factors” in addition to RpoD. The RpoD-related sigma factors can be sorted into four groups, with RpoD in Group 1 (34, 43) and the ECFs in Group 4. Bacteria may contain more than a hundred ECFs. RpoE of *Escherichia coli*, needed to combat envelope stress, is a paradigm for this family (30, 33, 44–46). ECFs have only conserved domains 2 and 4, enabling them to bind to their specific −35 and −10 region. Binding to RNAP stretches both domains for optimal contact with the promoter elements (34). As shown with RpoE, just one base is pulled out of the DNA-motif at the −10 site (47). Compared to RpoD, this makes transcription initiation by ECF sigma factors less efficient and in need of correctly positioned and better conserved −35 and −10 motifs in the promoter regions (34).

Groups 2 and 3 contain the starvation-stress sigma factor RpoS, heat-shock RpoH, and flagellum-synthesis factor FliA, which are all present in *C. metallidurans* and in *E. coli*. FliA contains domains 2, 3, and 4. Since domain 3 may interact with the extended −10 site, FliA should be able to use an extended −10 site in addition to the −35 site to bind and position the FliA-RNA-holoenzyme at a promoter. RpoS and RpoH contain domains 2, 3, and degenerate −35 region and 4, and additionally domain 1.2, allowing contact with the discriminator, which controls the final promoter escape of the RNAP (34). RpoS was derived from RpoD by a gene duplication and preferentially recognizes promoter motifs resembling those of RpoD. Differences between both consensus motifs include a YAaACT for RpoS instead of the TATAAT, an extended −10 region TttGC, a more degenerate −35 region, and an AT-rich discriminator between −10 and +1 (48, 49). The number of promoter elements in addition to the −35 site used by the different groups of RpoD-related sigma factors increases from the UP-Element and activators for ECF, plus the extended −10 for FliA, and finally the discriminator for RpoH, RpoS, and RpoD, which indicates an increasing ability to use promoters with weak or unfavorably positioned −35 site.

Sigma factor RpoN is not related to RpoD and its activator- and ATP-dependent transcription initiation process, as well as the promoter motifs are completely different from that mediated by RpoD (50). *C. metallidurans* and most other bacteria have one RpoN, while some nitrogen-fixing bacteria have two (31). Only RpoN is able to bind directly to DNA, albeit with just 10% efficiency compared to the RpoN-RNAP holoenzyme (51, 52). For all other sigma factors, direct DNA-binding is prevented, in RpoD by the sigma factor domain 1.1, in all RpoD-like alternative sigma factors by a compact structure of the free sigma factor (34).

An interesting question concerns the distribution of the transcriptional landscape among ECFs and other sigma factors (34). They could be orthogonal and specifically control the expression of their specific set of genes, or they may interact using multi-sigma factor hybrid promoters, hierarchies, or networks of sigma factors. The 11 ECF sigma factors of *C. metallidurans* are involved in metal homeostasis. Gene arrays using deletion strains failed to reveal their target genes but demonstrated that sigma factors in this bacterium interact and can substitute for each other (29). Moreover, no suppressor mutation could be identified that explained the upregulation of potential target genes in sigma factor mutants by other sigma factors (25). This led to the question of whether the 16 RpoD-like sigma factors exhibit an interplay or whether they are orthogonal with respect to metal homeostasis in *C. metallidurans*. To that end, transcriptional start sites (TSSs) were determined in metal-stressed and metal-starved cells, and these were linked to the sense and antisense transcripts determined in parallel in the same cells (26) to obtain a view of the transcriptional landscape of the bacterium. For all sigma factors, except RpoN, consensus motifs were derived, and the TSSs could be assigned to one or more sigma factors or ECF families. Unexpectedly, only RpoD-specific −10 and −35 motifs were found, while none for ECF sigma factors, FliA, or RpoH could be identified. Instead, a large group of promoters was identified with a correctly positioned −10 but incorrectly positioned −35 site, named “sba” for “sliding-blocking-another sigma factor” (27). These sba promoters may actually mask the consensus motifs of other sigma factors, under those for the more frequently occurring RpoD motifs. On the one hand, this could indicate that the RpoD-RNAP holoenzymes and the alternative sigma factors compete for promoter-binding, while on the other hand, RpoD-RNAP is ready as a back-up should the other sigma factor not be able to perform the required task. This indicates a significant dominance of RpoD for transcription initiation in *C. metallidurans*: RpoD is the one sigma factor that rules them all.

## RESULTS

### Metal-dependent transcriptional start sites in *C. metallidurans*

Transcriptional start sites (TSSs) were determined using the Cappable-seq-enrichment strategy (53) in metal-challenged, metal-starved, and control cells of *C. metallidurans* CH34 wild type, as well as its plasmid-free derivative strain AE104 in the same RNA samples that were used for measurement of the transcriptome (26). This allowed subsequent assignment of the TSSs to the sense and antisense RNAs, resulting in six data sets. Each was based upon three biological replicates. The 47,849 TSS signals appeared at least twice in one of them. They all were named according to their position, DNA strand, and replicon number, and displayed abundance scores between 14, just above the cutoff of 10, and 238,493 for TSS_2579185 + 3 upstream of *cspA*, encoding a cold-shock protein, which was present on the “+” strand at position 2,579,185 on the replicon number 3, the chromid.

The newly found TSSs were binned to those in the already published TSS data set for non-challenged *C. metallidurans* wild-type cells (27), totaling 15,758 TSSs that appeared in at least one of the seven data sets. Some of these TSSs have been previously published (26, 28, 54), but they have not been analyzed in detail or in context with other TSSs.

The scores and regulatory pattern of the 12,914 TSSs in the new data set were compared with each other (Table 1, crude data in File S4). Comparison was not done with the already published TSSs because their mean quality score was six times lower due to improved quality of the new TSS determination (Fig. S1). Nearly half of these approximately 13,000 TSSs were regulated under metal-stress conditions in strain CH34, but only 18% were regulated under metal-starvation. The percentage of metal-stress-regulated TSSs was higher on either plasmid compared to the chromosome or chromid, again highlighting the importance of these plasmids in metal homeostasis. With respect to TSSs specifically regulated in the comparison between the plasmid-free strain AE104 and its parent, CH34, 35% were regulated under metal-stress conditions, while a smaller portion of 17% and 18% was regulated in control, or in metal-starved cells, respectively. Each TSS was also provided with a tag (File S4) to indicate whether it was previously published, and if so, in which strain the TSS was found. A regulation tag indicated whether the TSS was regulated, the ratio between maximum and minimum score in the comparisons, plus the strain and condition having the highest abundance of the respective TSS. Every TSS with a tag of “Reg_3” or higher was judged to be regulated because it indicated a ratio of the maximum and minimum score of larger than 2.5.

**TABLE 1:** Number of transcriptional start sites (TSS) that were up- or down-regulated under various growth conditions in *C. metallidurans* strains CH34 or AE104^*a*^

Replicon	CH34: M/0	CH34: E/0	0: A/C	M: A/C	E: A/C	Total
Chrom.	3,041 (42%)	1,263 (17%)	1,420 (20 %)	2,885 (40%)	1,403 (19%)	7,198
Chromid	1,846 (42%)	766 (17%)	901 (20%)	1,677 (38%)	821 (19%)	4,389
pMOL30	547 (65%)	165 (20%)	n.p.	n.p.	n.p.	836
pMOL28	284 (58%)	96 (20 %)	n.p.	n.p.	n.p.	491
Total	5,718 (45%)	2,290 (18%)	2,321 (18%)	4,562 (35%)	2,224 (17%)	12,914

^*a*^ The number of TSSs is shown that are significantly (D > 1) up- (> 2-fold) or down- (< 0.5-fold) regulated on the respective replicons (Chrom., chromosome) in metal-stressed (M/0) or -starved (E/0) CH34 cells, or in the comparison with the control (0:), metal-stressed (M:), or -starved (E:) AE104 compared to CH34 cells; n.p., no plasmids in strain AE104 and therefore no comparison was possible. The overview of the regulated individual TSSs is in the Supplement.

These data demonstrated that most of the TSSs in *C. metallidurans* were regulated under conditions of changing metal availability, which resulted in a large re-organization of the transcriptome in this bacterium. This indicated a whole-physiology response to metal availability, broadly highlighting the importance of metal homeostasis to overall physiology.

### Impact of the transcriptional start sites on the sense and antisense transcripts

A transcriptional landscape for metal-stressed or starved *C. metallidurans* cells was constructed (File S1 in the Supplement). It contained for all elements such as TSS, the 5′ and 3′ untranslated regions (5UTR and 3UTR), the positions of antisense (AST) and sense transcripts (NPKM; NPKM values indicate the abundance of a gene transcript as nucleotide activities per kilobase of exon model per million mapped reads) in the published data set (26) for each DNA strand of all four replicons, their maximum abundance, their regulatory tag, and their impact, which were for either element the associated genes.

About 40% of the TSSs were associated with antisense transcripts, with the percentage increasing from 35% for TSSs on the chromosome to 48% on plasmid pMOL28 (Table 2). About 70% of the genes in *C. metallidurans* were not influenced by changing metal conditions (Table 2, lower part), while 30% were. However, of the TSSs associated with genes, 66% were regulated. This indicated a disparity: there was a comparably low number of unregulated TSSs associated (33.6% ± 5.2%) with unregulated genes (71.4% ± 2.3%), but a higher number of regulated TSSs (66.4% ± 5.2%) associated with regulated genes (28.6% ± 2.3%). Since bacterial genes may be arranged in multi-cistronic operons, non-regulated genes may be present in large operons with one promoter upstream of the operon, while regulated genes are in smaller operons, or have more than one transcriptional start site. Different changes in metal availability could affect the regulation of different groups of comparably small operons, for instance, those that were part of metal-resistance determinants.

**TABLE 2:** Percentage of transcriptional start sites associated with sense and antisense transcripts that were regulated or not by changing metal availability*^a^*

Replicon	TSS_AST	Not Reg.	Regul.	TSS_Sense	Not Reg	Regul.
Chromosome	2,228	20.4%	79.6%	4,181	27.9%	72.1%
Chromid	1,493	23.3%	76.7%	2,510	31.5%	68.5%
pMOL30	317	25.9%	74.1%	431	34.8%	65.2%
pMOL28	220	35.5%	64.5%	241	40.2%	59.8%
Mean		26.3%	73.7%		33.6%	66.4%
Deviation		6.5%	6.5%		5.2%	5.2%

Replicon	AST	Not Reg	Regul.	Genes	Not Reg	Regul.
Chromosome	2,050	31.3%	68.7%	3,870	74.4%	25.6%
Chromid	1,582	34.2%	65.8%	2,504	71.3%	28.7%
pMOL30	130	46.2%	53.8%	283	68.9%	31.1%
pMOL28	97	66.0%	34.0%	148	70.9%	29.1%
Mean		44.4%	55.6%		71.4%	28.6%
Deviation		15.7%	15.7%		2.3%	2.3%

^*a*^ The table gives in the upper part the number of transcriptional start sites on each of the four replicons and groups them into TSSs upstream of a transcript (AST) or not (Sense; 3UTR, 5UTR, genes), and whether the respective TSS was regulated (Regul.) or not (Not Reg). A TSS was considered to be regulated when the ratio of the highest divided by the lowest TSS score under the six (chromosome, chromid) or three conditions (plasmids, only in strain CH34) was ≥ 2.5 and subsequently rounded to 3, or a higher number. These values are compared in the lower part of the table with the number of ASTs, or genes (named NPKM) that are located on these replicons.

### Consensus motifs for RpoD- or RpoS-dependent promoters dominate the TSS upstream sequences

Possible promoter consensus motifs appearing between −90 and +10 of the TSSs were searched again using the published discovery software (27). The identified regions were subsequently scored with respect to the degree of conservation of the −35 and −10 sites (27).

**Fig 1:**
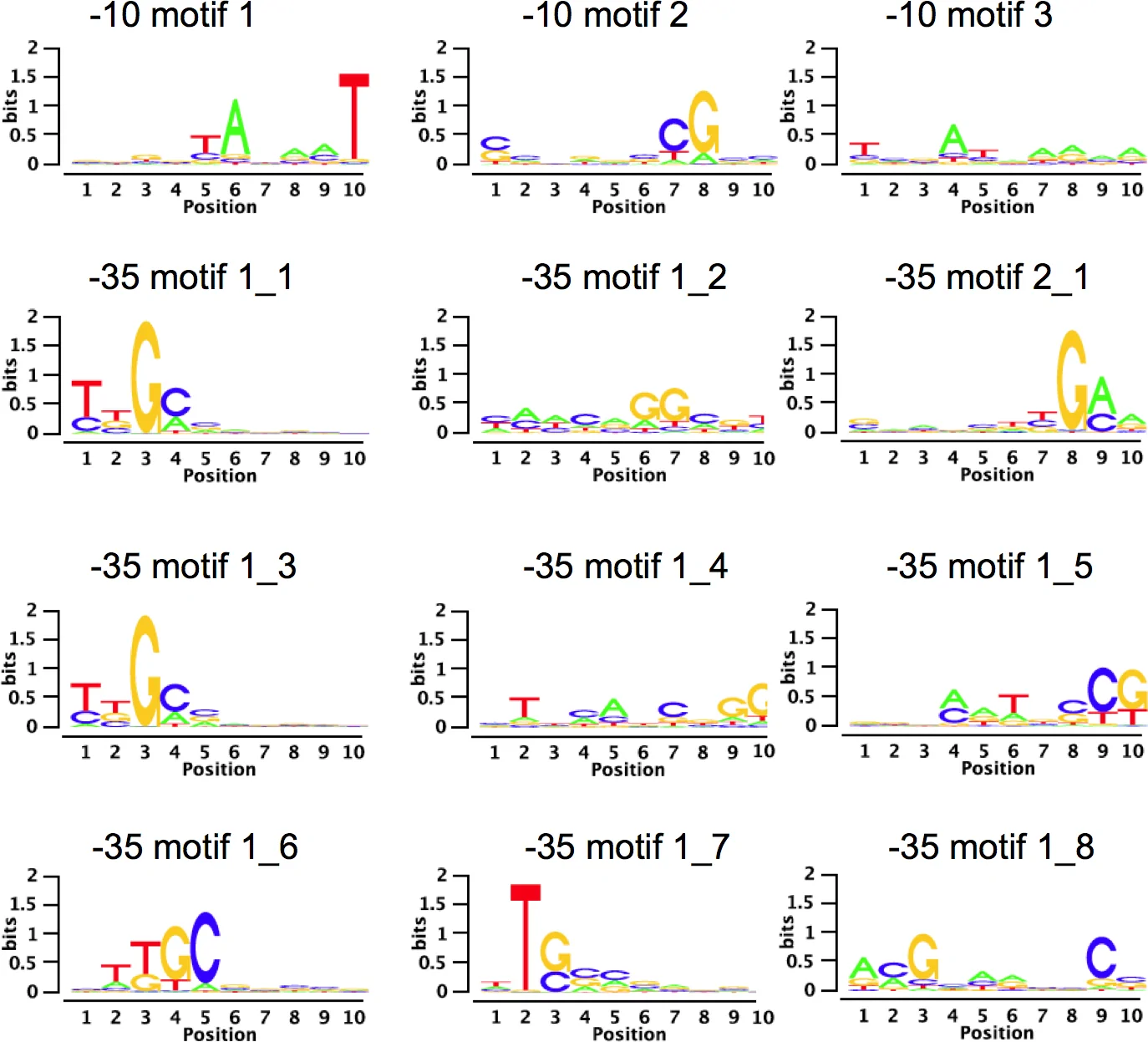
Overview of the motifs discovered in the regions upstream of TSS. For each motif, a sequence logo is shown. The −90 to + 10 regions of 12,914 TSSs were searched for patterns using the published custom-built discovery software based on hidden Markov models (27), which yielded the three −10 motifs on the top. The TSS with the −10 motif 1 were used in a similar search for upstream motifs, which yielded the −35 motifs 1_1 and 1_2. Likewise, the 226 TSS with the −10 motif 2 gave rise to the −35 motif 2_1. In a second approach, the TSSs that exhibited −10 motif 1 were sorted into RpoD, RpoS, and RpoDS subgroups, and −35 motifs were searched within these groups. For the RpoD subgroup, the −35 motifs 1_3 and 1_4 were found, for RpoS 1_5 and 1_6. A search independent from the −10 site gave motifs 1_7 and 1_8.

Among the TSSs identified here, 86.4% exhibited −10 motif 1 (Fig. 1). The −10 motif 1 displayed a clear preference for the positions at −13 and −12 for the first T, plus a minor group at −11 and −14, plus a few additional signals further upstream and downstream. The majority of these motifs had the prominent last T at position −7 or −8 upstream of the +1 of the TSS, agreeing perfectly with the value expected from the structure required for transcription initiation by RpoD-RNAP (38). Motif 1 also resembled the −10 motif TATAAT identified previously in *C. metallidurans*, with the second A and last T most prominent in promoters upstream of highly expressed housekeeping genes (27), which also agrees with the structure of the RpoD-RNAP during transcription initiation (38). This means that about 85% of the TSSs possessed a −10 motif for an RpoD-dependent RNAP in *C. metallidurans* cells. Two additional −10 motifs were found (Fig. 1), but neither had any positional preference. Most likely, these −10 motifs 2 and 3 represented background “noise,” and they were not further considered. Among the approximately 13,000 TSSs, no −10 motif appeared that was not binned with TATAAT.

**Fig 2:**
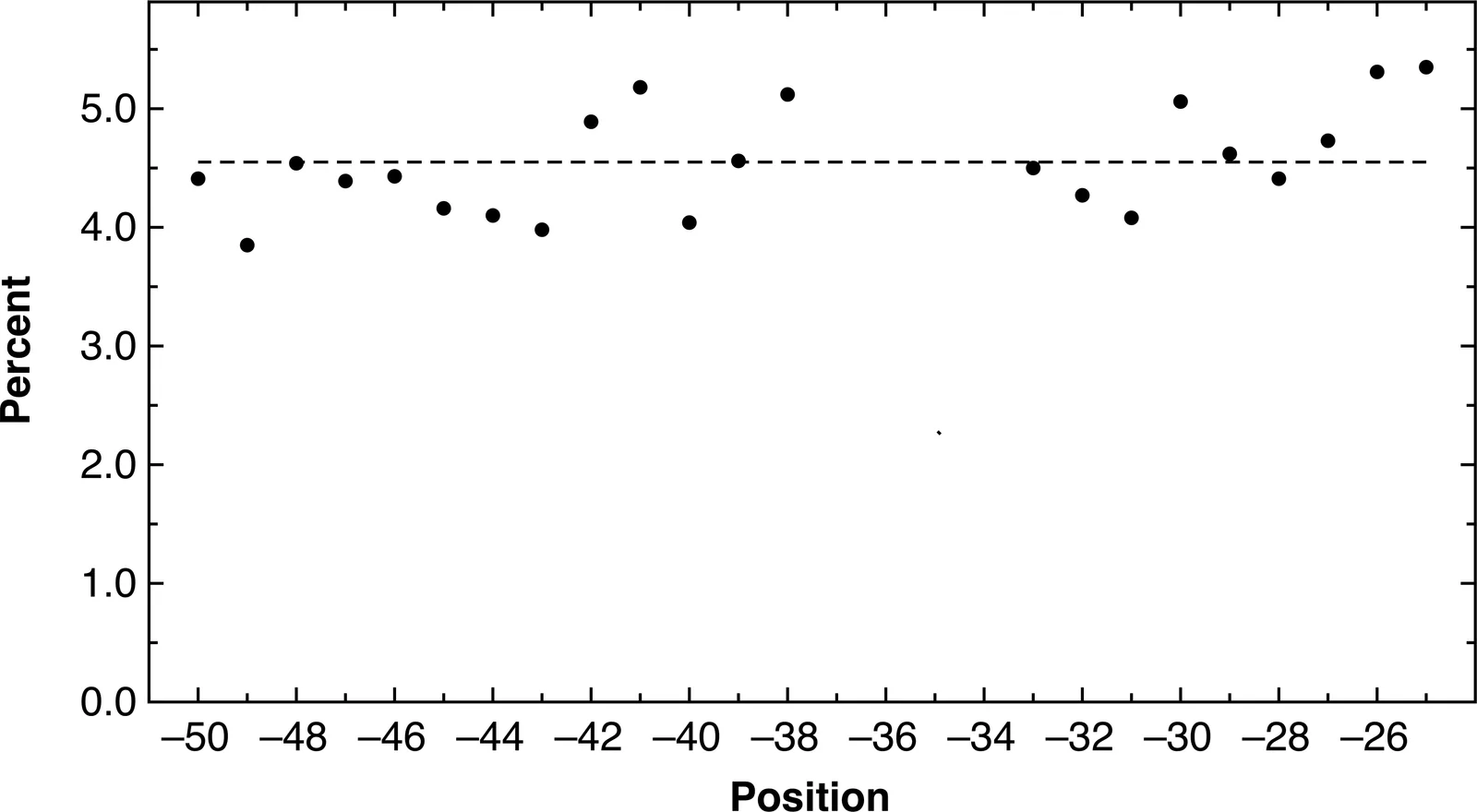
Distribution of the −35 motifs in sba (RpoD)-grouped promoter regions.

For the TSSs with −10 motif 1, their corresponding −35 motif was classified as group 1_1 and 1_2 (Fig. 1). The −35 motif 1_1 displayed a positional preference around −35 and −36, with additional positional preferences further upstream and downstream (Fig. 2). In contrast, −35 motif 1_2 exhibited no positional preference. From the consensus sequence and the position, −35 motif 1_1 represented a classic −35 consensus sequence TTGACA (41), also found in *C. metallidurans* (27).

It is possible that the consensus sequence for the starvation sigma factor, RpoS, may lie within the identified RpoD-like motifs. Differences between the extended −10, the −10, and discriminator consensus motifs (48, 49) were used to develop a scoring system for RpoS or RpoD motifs, grouping the TSSs with −10 motif 1 in either a RpoS, a RpoD, or a subgroup lying between both (RpoDS). In the subsequent pattern searches, the RpoD subgroup possessed the −35 motifs 1_3 and 1_4, with 1_3 again being similar to the TTGACA motif and showing a clear positional preference, which was not evident for −35 motif 1_4 (Fig. 1). The −35 motifs 1_5 and 1_6 of the RpoS subgroup also did not show a clear positional preference.

The −35 motifs were also searched independently of the −10 motif, leading to −35 motifs 1_7 and 1_8 (Fig. 1). Again, motif 1_7, but not 1_8, was similar to the motif TTGACA and displayed a positional preference at the −35 site. As in the case of −10 motifs, no other −35 motifs were found in addition to the TTGACA motif.

This indicated a huge dominance of the −10 and −35 consensus motifs for RpoD.

### Positions of the promoter motifs

Since the pattern search did not yield motifs other than the known RpoD −10 and −35 motifs, those of other sigma factors upstream of the TSSs might have been too rare to generate a pattern, but their consensus sequences might also overlap with, or be masked by, the RpoD/RpoS motifs. As done previously (27), candidates for −10 and −35 motifs were identified in the −90 to +10 regions of 12,814 of the 12,914 TSSs. The candidates were scored for conservation of the −10 and −35 RpoD consensus motifs; both values were added, and a penalty for incorrect positioning of these sites was applied.

When the −10 site was at the correct position, but not the −35 site, the promoter was labeled “sba” for “sliding-blocking-another sigma factor” as published (27). Surprisingly, these sba promoters included the majority of all RpoD/S promoter motifs (Table 3), and they were 2.5 times more abundant than promoter motifs with correctly positioned −10 and −35 sites. RpoD-like sigma factors may use, in addition to, or as a substitute for, the −35 site DNA-binding activators, the UP-element, an extended −10 site, or even the discriminator. Taking these possibilities into consideration, sba(RpoD) promoters could be fully functional promoters despite an incorrectly positioned −35 site because the four alternative factors might take over the task of RNAP positioning. The −35 RpoD motifs of sba(RpoD) promoters were in a ratio of 13:9 upstream and downstream, respectively, of the optimal position −35.5 (Fig. 2), so that the RpoD-RNAP complex would have to slide up or down the DNA for positioning using an alternative method. This would leave the ideal −35 position free for a consensus sequence of another sigma factor with a rarely occurring −35 site, which had to compete with the housekeeping RpoD-RNAP for binding. This means that sba(RpoD), sba(RpoS), or sba(RpoDS) indicates a promoter with lower efficiency compared to those with correctly positioned −35 and −10 sites, if other promoter elements or regulatory proteins do not compensate for the insufficient positioning of the −35 site, and that this promoter region might also be used by another sigma factor. In such a scenario, the −10 consensus motifs of alternative sigma factors might be hidden under the TATAAT. To address this possibility, all −10 motifs were scored separately and not combined with the −35 scores to reveal the second-best sigma factor able to bind the respective −10 sequence (File S3).

**TABLE 3:** Distribution of promoter motifs in transcriptional start sites of *C. metallidurans^a^*

Group	Total	%	Position OK	ba	sba	PosFail
RpoD	7,238	56.0%	1,737 (+114)	223 (+15)	4,341 (+256)	494 (+58)
RpoDS	2,953	22.9%	699 (+0)	106 (+0)	1,916 (+0)	232 (+0)
RpoS	604	4.68%	93 (+21)	19 (+1)	310 (+61)	81 (+18)
RpoH	393	3.04%	65 (+5)	14 (+2)	207 (+37)	45 (+18)
FliA	68	0.53%	5 (+4)	3 (+0)	22 (+18)	8 (+8)
ECF02	229	1.77%	32 (+6)	18 (+3)	104 (+18)	34 (+14)
ECF11	211	1.63%	26 (+6)	7 (+7)	92 (+19)	43 (+11)
ECF16	177	1.37%	9 (+1)	8 (+6)	70 (+8)	58 (+17)
ECF18	52	0.40%	1 (+1)	0 (+0)	16 (+7)	17 (+10)
ECF26	65	0.50%	1 (+2)	5 (+3)	14 (+5)	21 (+14)
ECF37	121	0.94%	11 (+0)	10 (+4)	45 (+10)	33 (+8)
ECF207	87	0.67%	9 (+0)	10 (+2)	30 (+2)	32 (+2)
ECF243	221	1.71%	10 (+5)	12 (+3)	83 (+14)	78 (+16)
ECF291	395	3.06%	54 (+0)	27 (+0)	201 (+0)	113 (+0)
Not assigned	100	0.1%				
Total	12,914					

^*a*^ The table gives the number of upstream sequences assigned to the consensus sequences of different sigma factors or ECF families. The number in parentheses is a multiple sigma factor assignment, indicating that a promoter motif was assigned to more than one sigma factor with a similar score.

When the −35 site but not the −10 site was correctly positioned, the promoter was grouped as “ba” (for “blocking-another” sigma factor) because an RNAP holoenzyme may be unable to form the open binary complex easily. When both promoter motifs were located at the wrong positions, the promoter was grouped into “PosFail” for “position failed.” In both cases, an RNAP holoenzyme might bind here and block access to an RNAP using another sigma factor that was able to initiate transcription here. “PosFail” might also indicate that the identified motifs were false positives and that the real promoter sequence responsible for initiation of transcription had not been identified.

The promoter-assignment (File S3 and File S1 in the Supplement) contains in the row “Sigma” either ba, sba, PosFail, or nothing (correctly positioned motifs), followed by the sigma factor and the motif score and final score in parentheses (motif score with position penalty subtracted). Should the TSS have been published (27), this information was added as “Pub” with the published grouping as strong, medium, weak, ba, sba, or non-RpoD-dependent promoter. Moreover, the name of the TSS, the distance to the closest gene, the maximum score, the regulatory tag, and the impact of the TSS were provided (TSS_Summary.xlsx and File S1 in the Supplement). The second-best sigma factor that might recognize the −10 or −35 motif was indicated for sba and ba promoters (TSS_Summary.xlsx).

### Dominance of RpoD/S-dependent promoters

More than half of the TSSs (56%) were assigned to RpoD (Table 3; total of 83.6% RpoD/RpoDS/RpoS), with 60% of the RpoD motifs grouped into “sba” and 24% with optimal −10 and −35 sites. Promoters grouped into “ba” or “PosFail,” in contrast, were in the minority, 3% or 7%, respectively, for RpoD promoters. Nearly 3,000 TSSs were in between RpoD and RpoS, indicating usage by both sigma factors. Only 604 seemed to be RpoS-dependent promoters. In the case of RpoDS and RpoS, the majority of these promoters were also sba promoters, followed by those with a correct position of the −35 and −10 site, plus a minority of ba and PosFail promoters.

To analyze the consequences of these assignments in more detail, a list of the promoters assigned to the 10 TSSs with the strongest scores in all experiments was compiled (File S5). For all promoters, the highest-scoring promoter driving expression of the gene encoding the cold-shock protein CspA had an abundance score of 238,493 and was categorized as “ba(RpoD).” The mean maximum abundance scores of the strongest TSSs were 146,722 ± 39,622, and most of them were categorized as RpoD/S or sba(RpoD/S). Promoter number 10 was the RpoD-dependent promoter *zntAp* in front of the gene encoding the main zinc-exporting P-type ATPase ZntA, which was fully upregulated in metal-stressed cells with an abundance >100,000 (File S5). The mean maximum abundance scores for RpoD/S and sba(RpoD/S) promoters were similar, with 105,539 ± 43,536 and 105,520 ± 22,905, respectively, so that these sba promoters were not different in the activity of the associated TSSs compared to those with a correctly positioned −35 site.

Among the RpoS-dependent promoters, the 10 with the highest maximum abundance had a mean value of 20,230 ± 19,476, showing a strong decline in the value from the top- to the lowest-ranking TSS. Genes that might be connected to starvation conditions were *copR_1_* for the plasmid-encoded response regulator of copper resistance, *corA_1_* encoding the secondary magnesium importer, *ppk* encoding the polyphosphate kinase, which, however, possessed several other promoters upstream, *greA* encoding a transcription elongation factor, *uvrC* encoding a part of the nucleotide-excision DNA-repair system UvrABC, and *phoR* encoding the central phosphate and magnesium sensor. This indicates a function of RpoS in controlling uptake of metal ions and phosphate, and resilience against disturbance of RNA synthesis and integrity of DNA (49, 55).

These data indicated that 83.6% of the TSSs in *C. metallidurans* were associated with promoters that were mainly used by RpoD, but some also, to an increasing extent, used by RpoS. Surprisingly, the majority of these promoters were sba promoters with a correctly positioned −10 site, but a −35 site up- or down-stream of the optimal −35 position.

The preceding chapter and the subsequent part of the results is an abbreviated version with additional data presented in an Extended Results part in the Supplemental material.

### Motifs recognized by other sigma factors

The dominance of sba-type promoters opened up the possibility that a −10 region might indeed contain overlapping motifs for two sigma factors, resulting in two TSSs that were fused during the binning into one. Alternatively, a rare consensus sequence could be masked by, or hidden within, the dominant TATAAT motif for RpoD. *C. metallidurans* contains in addition to RpoD/S and RpoN, the latter of which was not addressed here, a heat-shock sigma factor RpoH, a motility sigma factor FliA, and 11 sigma factors of the extracytoplasmic functions (ECF) family. As in many other cases, the 11 ECF sigma factors were composed of just domains 2 and 4, connected by a linker (Fig. S2 to S4) (33, 56). They were re-classified (Fig. S5). The predicted promoter sequences for these ECF families (Fig. S6) were obtained from the ECF Hub Database (56) and used to derive a scoring system to identify promoter motifs potentially masked by those for RpoD (Table S1; Table 4). This process used the ECF11 family as a control, which has no known representative in *C. metallidurans*. The ECF consensus sequences were combined with those of RpoD, RpoS (48), RpoH (57), and FliA (58) (Table S1; Table 4). Again, possible consensus motifs for the −35 and −10 promoter elements for all the ECF sigma factor families, RpoH and FliA, that appeared between −90 and +10 of the TSSs were searched using published discovery software, but the search ended without result. Evidently, these promoter elements are indeed too rare to form a pattern or are hidden by the dominant RpoD/S motifs.

**TABLE 4:** Sigma factors and consensus sequences^*a*^

Group	Sigma	−35	−10
ICF	RpoD	XTTGACA	GXTATAAT
ICF	RpoH	CTTGAAA	CXTATTTT
ICF	RpoS	GCTGACA	GCTAAACT
MDF	FliA	TAAAGTT	CCGATAAT
ECF02	RpoE	XTTGAAC	XXGTCTAA
ECF11	none	XGTGATC	XXCGTATA
ECF16	RpoQ	XAATTCG	XXGGTTAC
ECF18	RpoP	XGCATCC	XXCGTATX
ECF26	RpoL,M	XGGAATA	XXCGTTTT
ECF37	RpoR	XGTCAAC	XXCGTTAT
ECF207	RpoO	XGAATAA	XXGGTCTX
ECF243	RpoIJK	XAGAGCC	XXCCGGCX
ECF291	CnrH	XATACGC	XXCCCTCA

^*a*^ Except RpoN, the sigma factors of *C. metallidurans* are listed, which belong to the intracytoplasmic functions ICF, motility, and differentiation or the ECF group of RpoD-like sigma factors (31). The ECF sigma factors were newly assigned to ECF families (Fig. S2). The predicted promoter sequences for these ECF families (Fig. S2) were obtained from the ECF Hub Database (56) and are used to derive a scoring system to identify promoter motifs masked by those for RpoD (Table S1). The position of the second base of the listed −35 motif had a mean value of −35.6, with a distance of 15.8 base pairs. For scoring, the match of underlined bases was counted 1.5-fold, bold-faced ones were counted as 2-fold, and underlined and bold-faced ones were counted in 3-fold.

In a cross-test (Table S2), the highest score was always obtained for the specific consensus motif of a sigma factor. Moreover, the identified upstream sequences for the genes *rpoO, rpoP,* and *rpoL* (59) were indeed assigned to the respective sigma factors; however, no TSS could be assigned to these predicted promoters. The RpoD pattern also scored for RpoH and RpoS (Table S2), so that some cross-talk between these sigma factors could be expected. Similarly, consensus sequences assigned to sigma factors belonging to the groups ECF11 (no representative), ECF18 (RpoP), ECF26 (RpoL, M), ECF37 (RpoR), and ECF207 (RpoO) may form a promiscuous group of cross-talking sigma factors, as may the three ECF243 “iron” sigma factors RpoI, RpoJ, and RpoK. In contrast, FliA, ECF02 (RpoE), ECF16 (RpoQ), and ECF243 (CnrH) could be orthogonal.

The number of predicted promoters (Table 3) used by alternative sigma factors in comparison to the negative-control group ECF11 indicated that these assignments may contain false-positive results in the case of FliA and all ECFs except ECF291. The only representative of ECF291 in *C. metallidurans* is the plasmid-encoded CnrH, but some ECF291-assigned TSSs were regulated in the plasmid-free strain AE104 (File S5), so that another sigma factor may use the promoter consensus motifs associated with ECF291. This leaves the assignments to RpoD, RpoDS, RpoS, ECF291, and RpoH as valid, although the assignment to CnrH may not apply in this case.

As in the case of RpoD, the number of sba promoters was higher than that of those with correctly positioned −35 and −10 sites. The TSSs with the highest abundance scores for alternative sigma factors were also listed (File S5). Control of expression of *clpB* (60) by RpoH and of *flhD1* for a flagellar transcriptional activator by FliA agreed with the physiological functions of these sigma factors.

**Fig 3:**
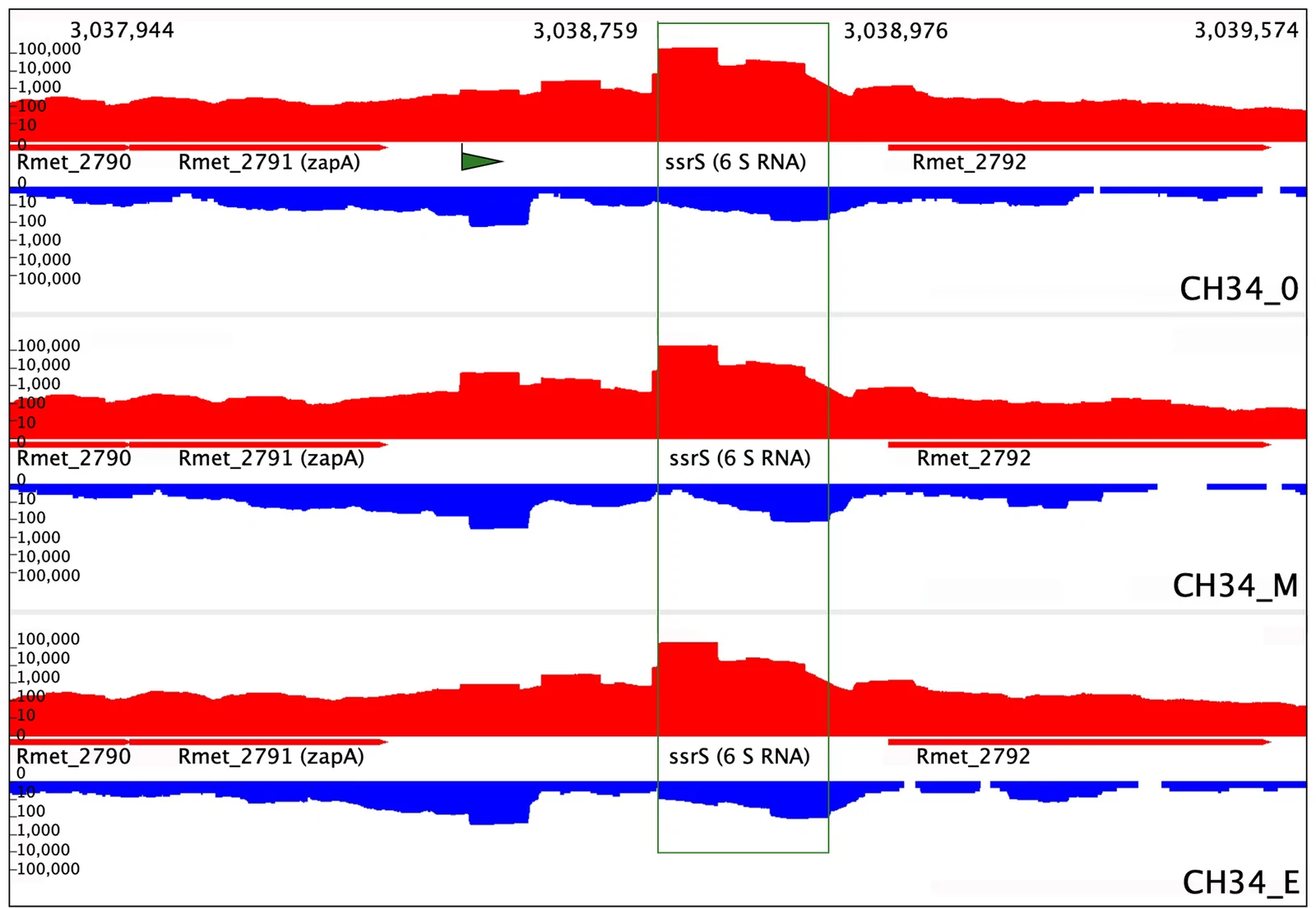
Region of the *ssrS* gene for the 6S RNA on the chromosome. The location of base pair 3,097,944–3,039,574 on the chromosome is shown with the transcript abundance in *C. metallidurans* CH34 control cells (CH34_0), metal-shocked (CH34_M), and metal-starved (CH34_E) cells, with the transcripts from the plus-strand in red and those from the minus-strand in blue. The *ssrS* region of the 6S RNA as predicted (61) is highlighted by the box. TSS_3038511 + 2 is shown as an arrowhead in the CH34_0 map, which is located 248 bp upstream of the predicted 5’ end of *ssrS*. The TSS has a maximum score of 81,591 in metal-shocked CH34 cells and is 14 times upregulated between the lowest and highest scores. Another TSS with a maximum score of 19 is not shown. TSS_3038511 + 2 had consensus sequences for the ECF243 family upstream, but was of the “sba” type with a correctly located −10 region and a not optimal −35 position. The “iron” sigma factors RpoI, RpoJ, and RpoK belong to this family.

The siderophore biosynthesis cluster that is under the control of RpoI (32) was not associated with ECF243. The TSS with the second highest abundance of 81,591 of all ECF243-associated TSSs was a sba(ECF243) promoter upstream of the gene *ssrS*, encoding the 6S RNA (Fig. 3), located between the gene encoding the cell division inhibitor, ZapA, and Rmet_2792 encoding a RNA-binding protein. The *ssrS* gene was expressed with high abundance in metal-challenged, metal-starved, or control cells of strain CH34 (Fig. 3). No difference in the intensity of the RT-PCR signal between exponentially growing and stationary-phase cells was evident (Fig. S7), and deletion of *ssrS* did not influence the growth of *C. metallidurans* (Fig. S8). This indicated that the 6S RNA in *C. metallidurans* cells was not essential, and its abundance did correlate with the growth phase, although a TSS upstream was upregulated under metal-stress conditions. This TSS may depend on an ECF243 sigma factor, such as RpoI, RpoJ, or RpoK.

### Metal homeostasis genes

**Fig 4:**
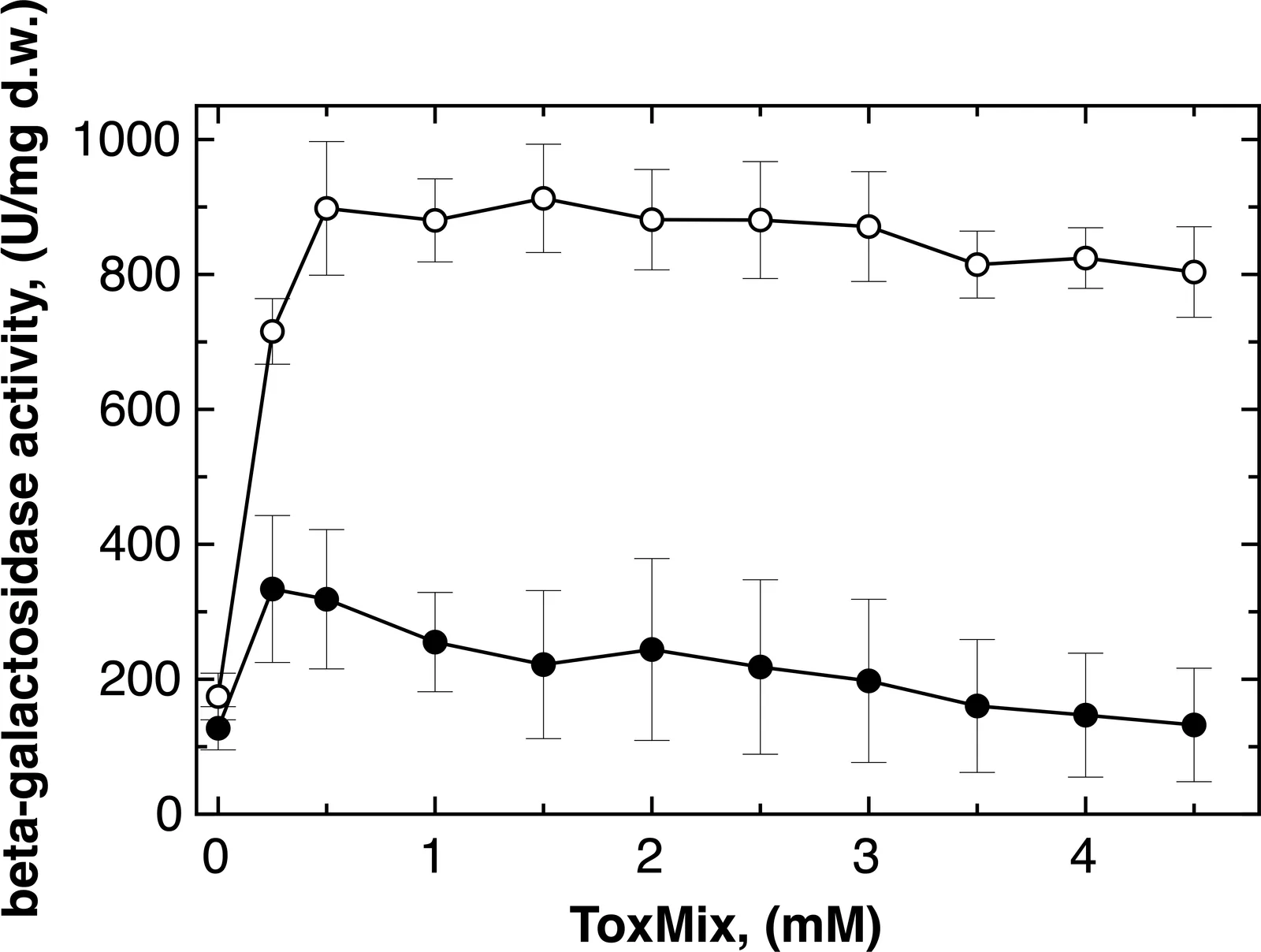
Comparison of the activity of promoter copA_2_ in *C. metallidurans* strain AE104 and *∆*rpoQ mutant DN658. *The cells contained a Φ*(copA_2_-lacZ) transcriptional fusion in strain AE104 (closed circles, L) and ∆*rpoQ* mutant strain DN658 (open circles, M). The strains were cultivated to the exponential phase of growth, AE104-specific ToxMix without arsenate was added, the cells were incubated with shaking at 30°C for 3 h, and the specific activity of the beta-galactosidase reporter was determined. Four independent biological experiments were performed, and deviations are indicated.

Using an intermediary data file (File S2), the promoters associated with the TSSs upstream of genes encoding gene products involved in metal homeostasis were extracted from the transcriptional landscape data (File S1). Moreover, the second-best sigma factors able to bind the respective −10 sequence or −35 sequence were also obtained (Table S3). The TSSs for most of these genes were under the control of RpoD or RpoS (Table 5). These data indicate a major role of RpoD in controlling the metal ion homeostasis of *C. metallidurans,* with some contribution made by RpoS, RpoH, and one or more sigma factors using the ECF291 consensus sequence; these represent promoters with mostly correctly located −35 and −10 elements, or sba promoters. A major exception, however, was a strong promoter responsible for the expression of the chromid-encoded *cop_2_* operon, which was assigned to ECF16 with RpoQ as the representative (Table 5). When the *copA_2_* gene was fused with *lacZ*, the reporter was upregulated by the ToxMix mixture of toxic metal ions in strain AE104 (Fig. 4); however, the expression was much stronger in the ∆*rpoQ* mutant strain. Contrary to expectations, RpoQ diminished metal-dependent upregulation of *copA_2_*. This indicated that the prediction of consensus sequences gave a positive result in this case, although not with the expected outcome.

**TABLE 5:** Overview of promoters^*a*^

Gene(s)	Promoter (bold main > 100, italics not regulated)
IM uptake	
*zupT*	RpoD; PosFail(RpoD); AST: sba(RpoD).
*pitA*	sba(ECF207); X_2_; *RpoD;* X_4_; RpoDS; AST: sba(RpoDS).
*corA1*	sba(RpoS).
*corA2*	*PosFail(RpoS);* AST: *sba(RpoD*).
*corA3*	*sba(RpoD)*.
*zntB*	X; sba(RpoD).
*feo*	RpoD; AST: sba(RpoD).
*mgtA*	X; *RpoS;*
*mgtB*	ba(RpoDS); PosFail (ECF291).
*pstSCABU*	sba(RpoD).
IM Efflux	
*zntA*	RpoD.
*cadA*	RpoD; X_2_; RpoDS; *sba(RpoD*).
*pbrA*	RpoD
*czcP*	RpoD; sba(RpoDS); AST: ba(ECF291); sba(RpoDS).
*cupA*	sba(RpoD); AST: ECF291.
*copF*	X; PosFail (RpoD); sba(RpoD). AST: sba(ECF37); *sba(RpoD*)
*ctpA1*	sba(ECF291); sba(RpoDS); sba(RpoD).
*rdxI*	sba(RpoD).
*czcD*	sba(RpoS); PosFail(RpoDS); sba(RpoD); AST: sba(RpoDS)
*dmeF*	X; sba(RpoD); sba(RpoD). AST: sba(RpoDS).
*fieF*	*sba(RpoD)*; RpoH. AST: PosFail(ECF207).
*cdfX*	RpoD.
*chrA_1_*	X; RpoDS.
*chrA_2_*	X_4_; sba(RpoD).
*arsB*	X_3_; sba(RpoD); AST: RpoDSECF291.
*atmA*	X2; RpoDS; *sba(RpoD*); sba(RpoD); RpoDS;
	AST: sba(RpoDS); *ECF291*.
Phosphate et al.	
*ppx*	sba(RpoD); sba(RpoD); sba(RpoD); *sba(RpoD)*; sba(RpoD).
*ppk*	sba(RpoDS); sba(RpoDS).
*gshAB*	sba(RpoD); *sba(RpoDRpoH*); RpoD.
*bfd, bfr*	RpoD.
*grxC*	sba(RpoD).
*cobW2*	X*; sba(RpoD)*.
*cobW3*	sba(RpoDS); sba(RpoD).
*cobW1* cluster	ba(RpoD); sba(RpoD); sba(ECF02); PosFail(RpoDS); sba(RpoD); PosFail(RpoD); *RpoDS*; *PosFail(RpoDS); sba(RpoD*).
Regulators	
*furA*	sba(RpoD);*sba(RpoD)*
*furB*	sba(RpoD); *RpoDS*.
*phoB*	sba(RpoD); AST: sba(RpoH)
*phoR*	RpoS; AST: sba(RpoH)
*phoU*	RpoD; sba(RpoD); AST: sba(RpoH)
*iscR*	PosFail(RpoDS); RpoD; AST: *sba(RpoD*); RpoDS.
*isc*	sba(RpoDS); AST: *sba(RpoD*); RpoDS.
*dksA*	PosFail (RpoS); sba(ECF26ECF207); sba(RpoS).
*zur*	*sba(RpoD)*.
*zntR*	sba(RpoDS).
*cadR*	PosFail (FliAECF243); RpoDS.
*pbrR*	RpoD.
*cupR*	sba(RpoD); AST: sba(RpoD); PosFail(ECF16); sba(RpoD).
*arsR*	sba(RpoD); AST: ECF11.
*czcRS*	sba(RpoD); sba(RpoD); sba(RpoD);
	AST: RpoDS; RpoD; PosFail(RpoD).
*czcR_2_S_2_*	PosFail (RpoD);*sba(RpoD*)
*copR_1_S_1_*	sba(RpoD)*^a^*; sba(ECF16); sba(RpoDS); sba(RpoS); *RpoD;*
	AST: sba(RpoD).
*copR_2_S_2_*	sba(RpoD); sba(RpoD); sba(RpoD); AST(*copR_2_*): ECF16
*zniCR*	sba(RpoD); AST: sba(RpoD)
*zniS*	*sba(RpoD);* sba(RpoDH); RpoD; sba(RpoD); sba(RpoD)
*zneP, zneR, zneS*	PosFail (ECF291).
*zneR_2_S_2_*	*sba(RpoDH).*
*hmzR*	RpoD.
*hmzS*	sba(RpoS).
Periplasmic Functions	
*czcN*	sba(RpoH); sba(RpoD); +3; AST: sba(RpoD), ba(ECF207).
*czcICBA*	sba(RpoD); two sba(RpoDS), RpoS, *sba(ECF16);* X; *sba(RpoD);* PosFail(RpoD), sba(RpoD); two sba(RpoD); AST(*czcIC*): sba(RpoD), sba(ECF11); *2 RpoD; RpoD*.
*czcA*	sba(RpoD); sba(ECF11); sba(RpoD); AST: 13 promoters.
*czcE*	sba(RpoDS); RpoD; sba(RpoD); AST: sba(ECF291).
*czcJ*	ba(RpoDS); sba(RpoDS); sba(RpoD); sba(RpoD); AST: +4
*czcI_2_C_2_B_2_'*	sba(RpoD); *zntA-*AST(*czcI_2_):* RpoD
*czcB_2_''A_2_*	*sba(RpoH*).
*ncc*	sba(RpoSECF243); RpoDS; PosFail (RpoDS)
*zneBA*	sba(RpoD); AST: sba(RpoD)
*zneC*	*sba(RpoDS*).
*zniBA*	RpoD; AST: sba(RpoDS); RpoDH; sba(RpoD).
*hmvCBA'*	PosFail(RpoD).
*hmvA''*	RpoD.
*hmyFCB*	sba(RpoD); PosFail (RpoD); sba(RpoH).
*hmyA*	PosFail(ECF16/18); sba(RpoD);
*nimBA_2_*	sba(RpoDS).
*nimA_1_C*	*sba(RpoD).*
*hmzBA*	RpoD; sba(RpoD); RpoD.
*cusDCBA*	*RpoD*; AST (*cusC*): three sba(RpoD); sba(RpoDS).
*cusF*	RpoDS.
*silDCBA*	sba(RpoD); *RpoD;* PosFail (ECF37); 4 ASTs/6 promoters.
*copQ*	sba(RpoH).
*copOFGJ*	sba(RpoD); 11 promoters; AST(*copO):* six promoters
*copN*	sba(ECF37); +2 promoters.
*copTV*	sba(RpoDS); sba(RpoD); +4 promoters.
*copM*	PosFail (ECF02); sba(RpoD); AST: sba(RpoD); sba(RpoD)
*copK*	RpoD; sba(RpoD); RpoDS;
*copA_1_B_1_C_1_D_1_I*	ECF02; sba(ECF11); RpoH; RpoD; sba(RpoD); *sba(RpoD);*
	AST: sba(ECF02); sba(RpoD); PosFail(RpoDS).
*copL*	sba(RpoD).
*copEW*	RpoD; sba(RpoDS); ba(RpoDECF11); ba(RpoD); AST: +4
AST(*copR_2_)-copA_2_B_2_C_2_D_2_*	ECF16;
*cnrYXHCBA*	sba(RpoD); sba(RpoDS); sba(RpoD); sba(RpoD);
	sba(RpoD); AST(*cnrA*): sba(RpoD); *sba(RpoDS);* RpoD; AST(*cnrB*): two sba(RpoD); RpoD; AST(*cnrXH*): sba(RpoD).

*cnrA*	sba(ECF207).
*cnrT*	*sba(RpoD);* two sba(RpoD); sba(RpoD); RpoD;
	AST: three sba(RpoD).
Sigma factors	
*rpoD_1_*	PosFail(ECFs)*^b^*; sba(RpoD);X;*sba(RpoDS);* AST: sba(RpoD)
*rpoD_2_*	sba(RpoD); *RpoD; sba(RpoD);* AST: six promoters.
*rpoS*	sba(RpoD); RpoD; *RpoD;* +4; AST: ECF11; +2 promoters
*rpoH*	sba(RpoDS); PosFail (RpoDS); AST: sba(RpoDS).
*fliA (Op1026f_3*)	X_5_; RpoD; X; sba(RpoD).
*rpoI*	X; RpoD.
*rsiA, Op0320r_2*	sba(RpoD); X; RpoD.
*rpoJ, rsjA*	sba(RpoD).
*rpoK, rskA*	X; *sba(RpoD);*
*rpoL, rslA, yedYZ;*	X; *PosFail (RpoDS),* sba(RpoDSECF243), RpoS.
*rpoM, rsmA, Op1608f*	*sba(RpoD).*
*rpoO, rsoA*	sba(RpoDS; *2427 bp to rpoO*).
*rpoE, rseA, rseB*	*RpoD; sba(RpoD);* AST: 11 promoters
*rseA, rseB*	sba(ECF291); AST: 11 promoters
*cnrYXHCB*	sba(RpoD); two sba(RpoD); two sba(RpoD); AST: sba(RpoD)
*rpoP, rspA*	*sba(RpoD);* PosFail (RpoDS); X; sba(RpoS).
*rpoQ, rsqA, gig*	PosFail (ECF02); AST; sba(RpoDS); AST: RpoH
*rpoR, rsrA, rsrB*	Sba(RpoH); RpoD; +6 promoters.

^*a*^ The predicted promoters upstream of the TSSs for the respective genes or operons were transferred from the Supplementary Material (Landscape.xlsx) as Regions.xlsx (Supplement). From here, the promoter association was used, and the promoters were listed from the most proximal to the most distal. “X” indicates additional open reading frames between the promoter and target gene in a multi-cistronic operon. Underlined is the responsible promoter of the gene or its operon. This is the most proximal promoter with the highest score. Bold indicates the promoters with a TSS score > 100. *Italics *indicate a TSS that was not regulated under changing metal availability (Reg_2 and NONE)*.* Gray fields indicate a possible contribution of an alternative sigma factor. Promoters for antisense RNAs covering the respective gene are also indicated if the AST score > 10. Table S3 supplements these data for the sba and ba promoters with the association of the –10 or –35 sequence, respectively, and with the second-best alternative sigma factor. The promoters were listed starting with the most proximal promoter from left to right.

The accompanying Extended Results part ends here.

### Sigma factors

The genes for sigma factor were also mostly under the control of RpoD or sba(RpoD/S) promoters. In most cases, the TSS that was identified could not be associated with the consensus motifs for the sigma factor that was synthesized as a product of the respective operon, except in the case of RpoD. The second exception was that expression of the genes encoding the anti-sigma factors RseA and RseB, which were assigned to a sba(ECF291) promoter and within the complete *rpoE-rseAB* operon, was under RpoD control. The third exception included a PosFail(ECF02) promoter, which was responsible for expression of the *rpoQ-rsqA-gig* operon via TSS_1315237 + 3 (Table 5; File S2).

**Fig 5:**
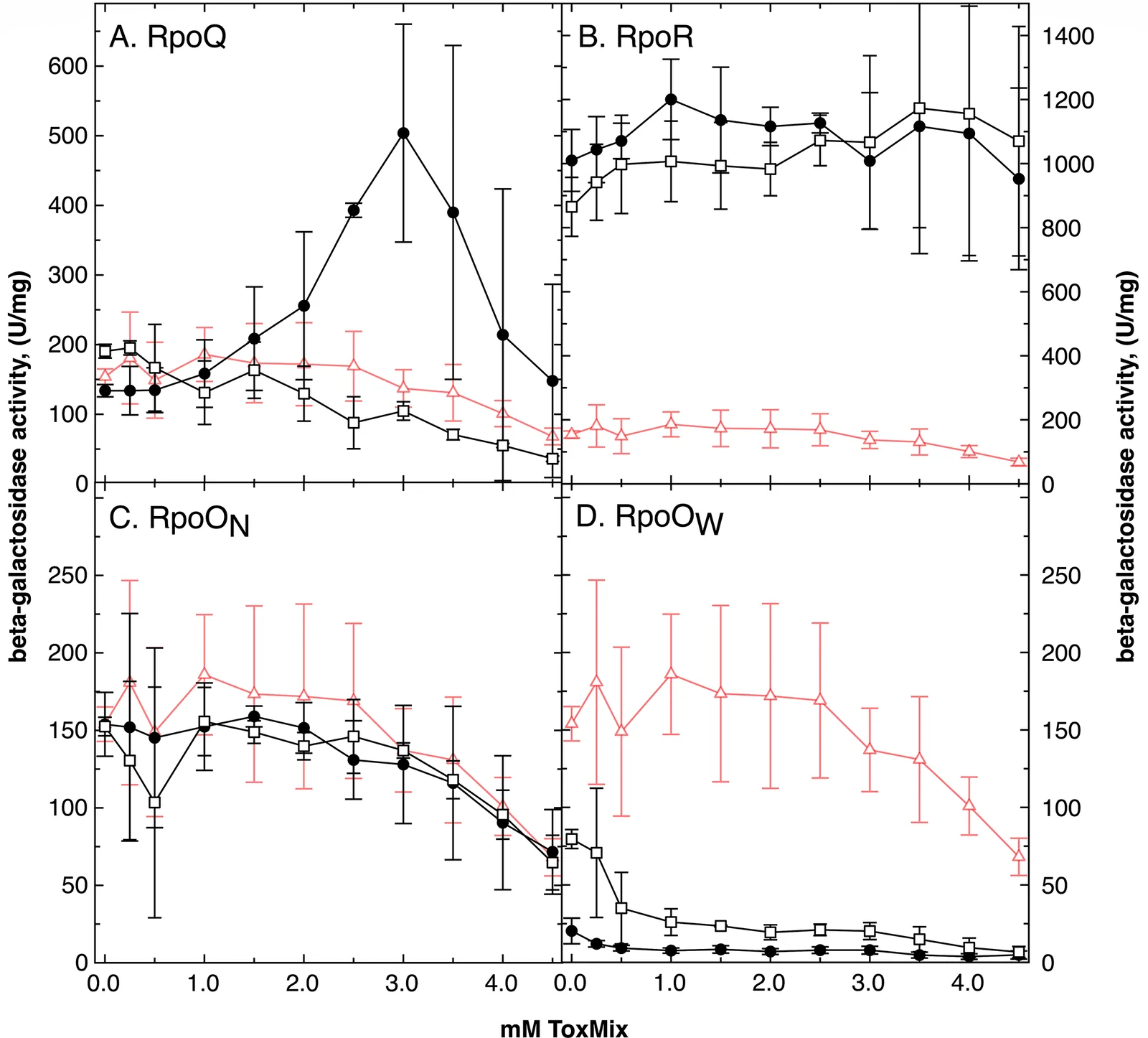
Comparison of the activity promoter regions in *C. metallidurans* strain AE104 and a mutant with the deletion in the gene for the respective sigma factor. The respective promoter fragments were cloned upstream of a promoter-less *lacZ* gene on plasmid pVDZ’2. The resulting plasmids were conjugated into strains AE104 (closed circles, L) and a mutant strain in the gene for the respective sigma factor (open squares, O). The sigma factors were RpoQ (Panel** A**), RpoR (Panel **B**), a region directly upstream of *rpoO* (RpoO_N_, Panel **C**), or 2.4 kb upstream of this gene (RpoO_W_, Panel **D**). The negative control was strain AE104 with the promoter-less *lacZ* gene on plasmid pVDZ’2 (light open red triangles, R). The conjugants were cultivated to the exponential phase of growth, AE104-specific ToxMix without arsenate was added, the cells were incubated with shaking at 30°C for 3 h, and the specific activity of the beta-galactosidase reporter was determined. The results of ten independent biological experiments are shown, and deviations are indicated.

The −35 and −10 sites upstream of TSS_1315237 + 3 for *rpoQ* had no similarity to the ECF16 consensus motifs but were nonetheless categorized as PosFail(ECF02). This region was cloned upstream of a promoter-less *lacZ* gene. In *C. metallidurans* strain AE104, this resulted in a strong metal-dependent upregulation of the expression of the reporter gene, which was absent in the ∆*rpoQ* mutant (Fig. 5A). The RpoQ consensus motifs were too rare to be found as a pattern and appeared to be masked under ECF02 motifs with incorrect positions. On the other hand, the *copA_2_p* promoter that was associated with the RpoQ group ECF16 was inhibited in cells containing RpoQ (Fig. 4), suggesting that RpoQ-containing RNA polymerase holoenzyme (RpoQ-RNAP) should bind here.

In the case of RpoO, RpoP, and RpoL, promoter motifs were predicted upstream of adjacent genes (RpoO and RpoP) or of the operon containing the *rpoL* gene (59). Indeed, these promoter motifs scored correctly for an assignment to the respective sigma factor (Table S2), but in no case could a TSS be identified directly downstream of the predicted −10 site. Instead, the identified TSSs were associated with RpoD and RpoS, and in one case, a TSS further upstream of the *rpoL*-containing operon RpoD, RpoS, or ECF243 was identified (Table S2). An autoregulatory process of the ECF sigma factors could not be derived from the data.

When the upstream region of *rpoR*, which was associated with a clear RpoD-dependent promoter, was cloned upstream of the promoter-less *lacZ* gene (Fig. 5B), this gene was expressed to a similar level in *C. metallidurans* strain AE104 and in its isogenic ∆*rpoR* derivative, providing corroborating evidence that the respective TSS was indeed controlled by a RpoD-dependent promoter. For *rpoO,* no TSS was found directly upstream of the gene, but a sba(RpoDS) promoter nearly 2.5 kb upstream was identified as the most proximal TSS under the conditions tested (Table 5). When both regions were cloned upstream of *lacZ*, the fragment directly upstream of *rpoO* showed only the activity at a similar level to that of the negative control (Fig. 5C, RpoO_N_). Unexpectedly, the region 2.5 kb upstream (Fig. 5D, RpoO_W_) strongly downregulated expression of the reporter gene, in metal-challenged cells more than in those with no ToxMix added, and this downregulation was partially relieved in the ∆*rpoO* mutant. This suggests that RpoO-RNAP likely binds at this sba(RpoDS) promoter and downregulates it. This is reminiscent of RpoQ-RNAP-dependent control of the *cop_2_* ECF16 promoter.

**Fig 6:**
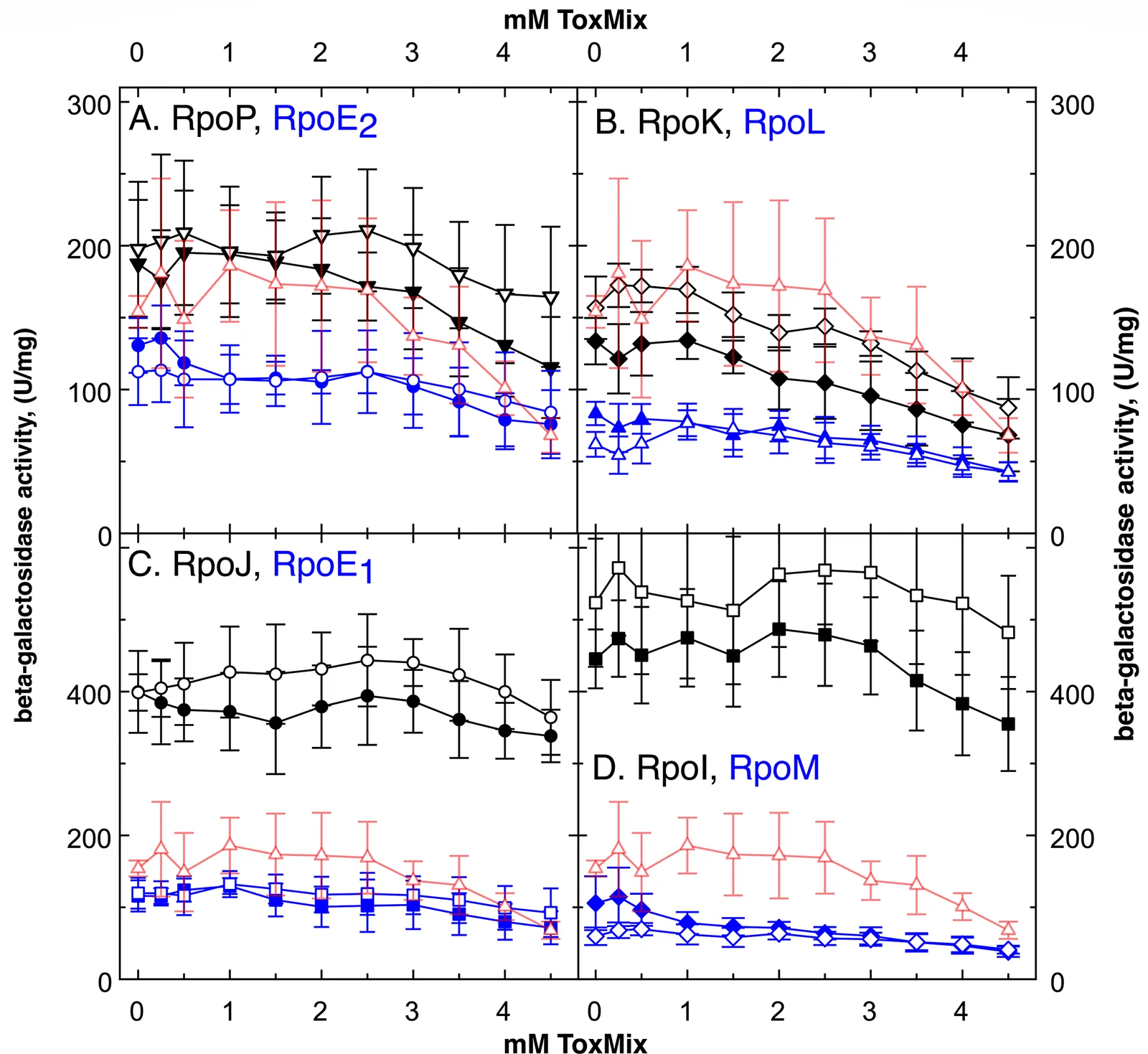
Comparison of the activity promoter regions in *C. metallidurans* strain AE104 and a mutant with the deletion in the gene for the respective sigma factor. The respective promoter fragments were cloned upstream of a promoter-less *lacZ* gene on plasmid pVDZ’2. The resulting plasmids were conjugated into strains AE104 (closed symbols) and a mutant strain in the gene for the respective sigma factor (open symbols). These were in Panel A, RpoP (inverted triangles s,q), and RpoE2 (sba(ECF291) promoter 388 bp upstream of *rseA*, blue circles m,l), in Panel B, RpoK (diamonds ¯,u) and RpoL (blue triangles r,p), in Panel C, RpoJ (circles m,l) and RpoE1 (RpoD promoter 11 bp upstream of *rpoE*, blue squares o,¢), and in Panel D, RpoI (squares o,¢) and RpoM (blue diamonds ¯,u). The negative control was strain AE104 with the promoter-less *lacZ* gene on plasmid pVDZ’2 (light open red triangles, R). The conjugants were cultivated to the exponential phase of growth, AE104-specific ToxMix without arsenate was added, the cells were incubated with shaking at 30°C for 3 h, and the specific activity of the beta-galactosidase reporter was determined. The results of ten independent biological experiments are shown, and deviations are indicated.

The promoter regions upstream of the genes for other ECF sigma factors were also cloned upstream of the promoter-less *lacZ* gene, and expression of the reporter activity was measured under metal stress conditions (Fig. 6). Three groups of results were determined. The first group (RpoP and RpoK; Fig. 6AB, black), associated with sba(RpoD) promoters, had activities in the range of the negative control and thus lacked expression. The activity was, however, higher in the respective sigma factor mutant. The second group (RpoJ and RpoI; Fig. 6CD, black), which was associated with RpoD and sba(RpoD) promoters, showed activity above the level of the negative control, and similarly to the first group, it had a higher activity in the respective promoter mutant. In these cases, this may indicate binding of, and repression by, the respective sigma factor-containing RNAP. Unfortunately, although measured 10 times, the reproducibility was too variable to allow a significant conclusion to be drawn such that these results must be taken with caution.

The third group (Fig. 6 in blue, RpoE promoters, RpoL, and RpoM) had activities below the level of the negative control, indicating repression. These promoters were categorized as PosFail(RpoDS) in the case of the *rpoL* promoter, sba(RpoD) for *rpoM*, RpoD for *rpoE-rseAB,* and sba(ECF291) for *rseAB* (Table 5). In the case of RpoE2 for *rseAB*, RpoL, and RpoM, the level of repression was stronger in the respective sigma factor mutant under conditions of low metal stress; however, this was again not a significant result due to high levels of deviation, despite numerous repeats.

In the case of the promoter upstream of *rpoQ*, motifs that were different from RpoD promoter motifs, and which were also not at the correct positions, suggested a clear RpoQ-dependent phenotype. In a second example, an ECF16-promoter was associated with a downregulation of expression by RpoQ. This downregulation was also visible, albeit with significant standard deviation of the respective experimental repeats, with regions upstream of other ECF sigma factors.

## DISCUSSION

### TSSs assigned to sba promoters

The intention of this work was to construct a complete transcriptome landscape in metal-stressed and metal-starved cells of *C. metallidurans* CH34 wild-type and its plasmid-free mutant AE104 to assign the identified TSSs to the sigma factors of this bacterium and subsequently to understand exactly how each individual sigma factor mediates the measured physiological effects (29, 31, 32). The changes in this transcriptome landscape demonstrate how significant the metal response of the bacterium is. While obviously, it is well appreciated that this organism is highly adapted to heavy metal conditions; nonetheless, it should be emphasized that around 70% of the TSSs were regulated by high or low metal conditions. This is a remarkably high number, indicating a whole-physiology response to metal availability, highlighting the importance of metal homeostasis to the physiology of *C. metallidurans*.

While the transcriptome landscape comprising the regulation and impact of 12,914 TSSs could be successfully constructed and the majority of these TSSs could be assigned to RpoD or RpoS, assignment to the ECF sigma factors, RpoH or FliA, could not be accomplished with the required fidelity. In the pattern searches, only the −10 and −35 motifs of RpoD were found, while those done with the motifs of ECF, RpoH, or FliA promoter elements yielded no clear result. In the case of the ECFs, this can be easily explained by the scarcity of operons and promoters under the control of a single ECF (47), but more TSSs should theoretically have been assignable to FliA and RpoH.

**Fig 7:**
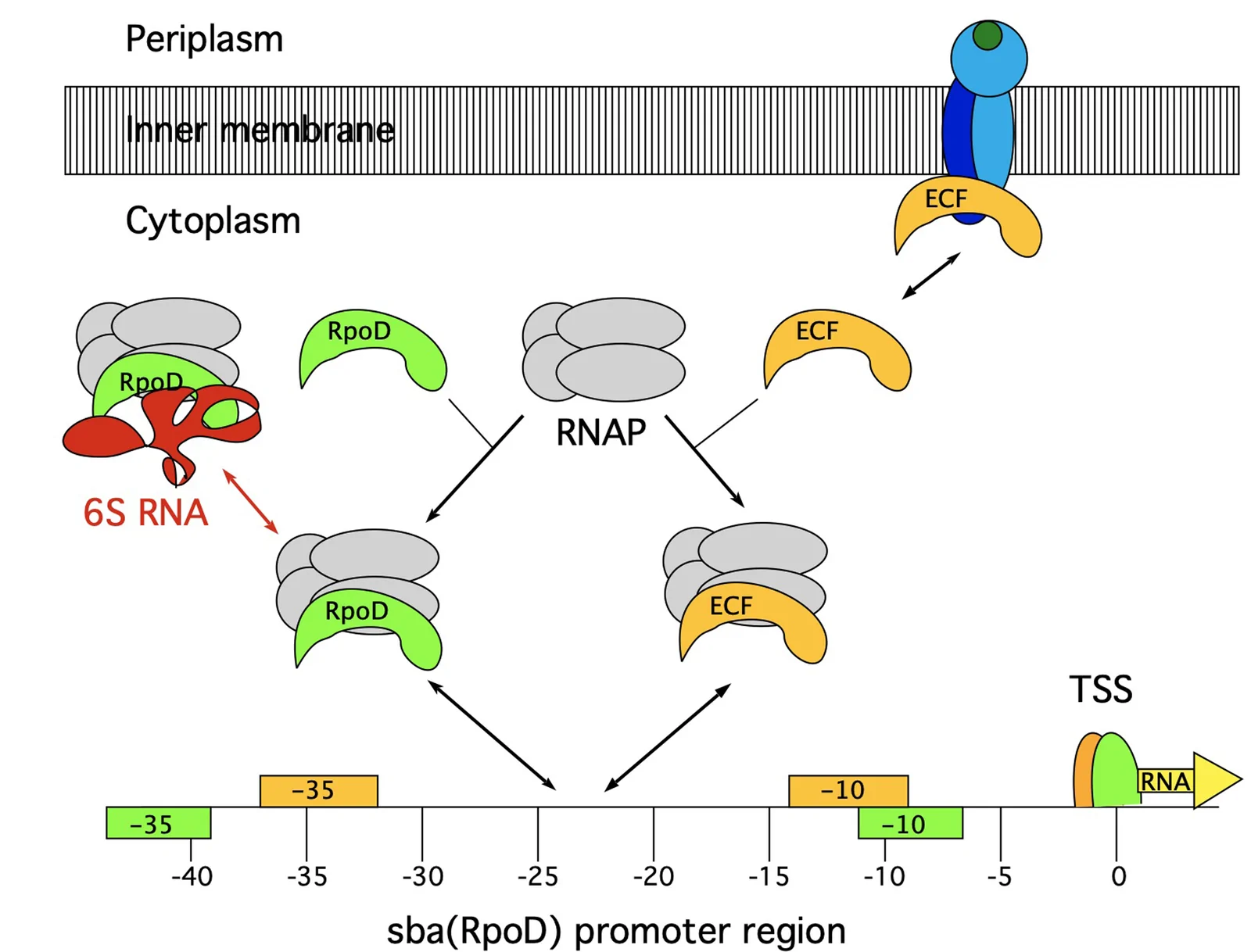
Model of a sba(RpoD) promoter site. The figure shows competition between RNA polymerase holoenzymes at a sba(RpoD) promoter, for example, between RpoD- and CnrH-RNAP holoenzymes. The sba(RpoD) promoter region is at the bottom with the –35, –10, and TSSs resulting from the transcription initiation events shown in orange and light green, respectively, and the RNA is shown in yellow. Please note that the typical sba(RpoD) promoter with a correctly oriented −10 element is shown, but the −35 site is too far upstream (in other examples, too far downstream). The −10 elements for both RNAP holoenzymes overlap, leading to overlapping TSSs that are, however, very close to each other and binned into one TSS by the data processing algorithm (Gaussian curves in orange and green above position 0). The two competing RNAP holoenzymes are shown above the promoter region, with RNAP in gray, RpoD in light green, and the other sigma factor, such as an ECF sigma factor, FliA or RpoH, in orange. Both compete for the RNAP core enzyme. While RpoD-RNAP might be sequestered by the 6S RNA (red) and removed from competition, the competing sigma factor might be degraded (RpoH), bound to a soluble anti-sigma factor (FliA), or a membrane-bound anti-sigma factor (ECF). As an example, the membrane-bound anti-sigma factor complex CnrYX is shown, which sequesters CnrH but releases this sigma factor when periplasmic Ni(II) (dark green) is bound to CnrX. Please note that the RNAP is actually much larger than shown here and covers the indicated DNA region.

The RpoD motifs may have been masking the motifs for the other sigma factors. These latter motifs may be annotated to the RpoD motifs and hide the rare motifs of the other sigma factors, or these motifs may overlap with the RpoD motifs. The possibility that the motifs of the other sigma factors were annotated to the RpoD motifs was tested (Table 3) and did not provide much insight. To aid motif identification, all TSSs within a distance of 5 base pairs up- or down-stream of the position with the highest TSS abundance were binned as one TSS. Due to this limited resolution, two −10 elements overlapping by five base pairs or less would lead to two TSSs binned into one, so that overlapping −10 elements could not be resolved by the method used. The presence of the promoter motifs for alternative sigma factors not overlapping with those used by RpoD, however, should result in a TSS not binned with the TSS assigned to the RpoD motifs. This means that an overlap of the −10 elements for RpoD and an alternative sigma factor might explain the results obtained (Fig. 7).

All three possibilities, “masking,” “annotation,” and “overlap,” imply that RpoD-RNAP and RNAP with an alternative sigma factor might use a hybrid promoter with overlapping −10 elements. This suggests that sigma factors not only compete for the RNAP core enzymes (62) but also for promoters, when complexed with RNAP as holoenzymes. The sba(RpoD) promoters should be ideal to allow such a promoter competition. The RpoD-holoenzyme could be attracted to such a hybrid sba promoter using the −35 site, an UP element, or through regulatory proteins to position it at the −10 sites (34, 39, 42). Alternative sigma factor-containing holoenzymes bind their −35 site, which was not revealed by the pattern search because the algorithm found only the highly abundant RpoD −35 element in the vicinity. Subsequently, transcription starts at a slightly different position compared to the TSS resulting from the RpoD-RNAP, but the positions of the TSSs would be so close to each other that they were binned into one TSS. The existence of sba promoters in combination with the observed sigma factor-dependent down-regulation (Fig. 4 to 6) supports such a scenario (Fig. 7).

In principle, some promoters that tend toward being classified as sba promoters have been known for a long time. For example, promoters regulated by the mercury-resistance regulator MerR usually have a distance of about 19 bps between the −35 and −10 site (63, 64), which would not allow efficient transcription initiation. The MerR regulator binds between both promoter positions. When Hg(II) is sequestered by the regulator, it changes its conformation, and consequently that of the DNA around the promoter, such that the −35 and −10 sites attain an optimal separation to allow transcription initiation. The promoters of the *mer* operons in *C. metallidurans* were actually annotated as ba or sba promoters. The promoters for *zntA, cadA,* and *pbrA,* which are under control of the MerR homologs ZntR, CadR, and PbrR, respectively (20, 65, 66), were actually under RpoD control, while *cupA,* under control of another MerR homolog, was classified as sba(RpoD) (Table 5). While *zntAp* has perfectly spaced −35 and −10 sites, *cadAp* and *pbrAp* had only a small distance penalty (File S1). This indicated that some promoters that interact with MerR-type regulators have a −35 element that lies a few base pairs upstream of the ideal position and could indeed be regarded as a first example of a sba promoter. However, in other cases, the DNA distortion caused by regulator binding may not be required for transcription initiation. The reason for this difference could simply be the much higher toxicity of Hg(II) compared to Zn(II) (67), so that MerR should act at a much lower metal concentration compared to ZntR.

### Transcription of the *cnr* determinant

Examination of transcription initiation at the *cnrYp* promoter provides evidence for the hypothesis that sba promoters might actually represent hybrid promoters recognized by RpoD and another sigma factor. The *cnr* determinant (Table S4) on plasmid pMOL28 encodes resistance to Ni(II) and Co(II), mediated by the transenvelope efflux system CnrCBA, which transports the substrate ions from the periplasm to the outside (68). Additionally, CnrT exports Ni(II) from the cytoplasm to the periplasm. Expression of *cnr* is up-regulated by Ni(II) (69, 70). The ion is sensed in the periplasm by the CnrX component of the anti-sigma factor complex CnrYX, which subsequently releases the sigma factor CnrH to initiate transcription of *cnr* (71–77). The TSSs upstream of cnrY and three of those located within cnrA upstream of *cnrT* were also determined in the published RNASeq study with non-challenged C. metallidurans cells (27) and previously by primer-extension studies (69). The highest-scoring TSS_53286 + 5 23 bp upstream of *cnrY*, designated *cnrYp*, was associated with the promoter model of RpoD in the previous study (27). On the other hand, nickel-dependent upregulation of *cnrYXHCBA-lacZ*, conferring nickel-resistance, strictly depends on the ECF sigma factor CnrH (69, 70). Consequently, it would have been expected that additional CnrH-dependent TSSs may appear or were upregulated by nickel exposure in metal-challenged cells. But this proved not to be the case. Neither *cnrCp* nor another TSS upstream of *cnrY* appeared in metal-challenged *C. metallidurans* cells (Table S4). Instead, promoter *cnrYp,* now annotated as a sba(RpoD)-promoter due to an improved analysis of the promoter elements upstream of the TSSs, was responsible for the highest-scoring TSS_53286 + 5 within the *cnr* region. It was upregulated 11-fold to a score of 28,233. A TSS 206 bp upstream of *cnrY* was upregulated 16-fold to a score of 82, but with the highest score in non-challenged cells. This TSS had also been found in non-challenged cells, but only in two of the three repetitions, and was not further considered (27). It was not upregulated by nickel and should consequently not be CnrH-dependent. A TSS 889 bp upstream of *cnrY* was below the threshold and the score of 25 is too low to explain the upregulation of the *cnr* genes. Two remaining TSSs more than 900 bp upstream of *cnrY*, one published (27) and one unpublished, were upregulated by metal stress but possessed a much lower score than TSS_53286 + 5 (Table 3). The published TSS_52343 + 5 was annotated as a sba(RpoD) promoter and would have been a candidate for the CnrH-dependent promoter. However, if that were the case, its TSS should have a higher score in the presence of nickel ions, compared with TSS_53286 + 5 (Table 3), but its score was 23-fold lower.

Since no potentially CnrH-dependent transcriptional start sites appeared in metal-stressed *C. metallidurans* cells, TSS_53286 + 5 directly upstream of *cnrY* must be the product of the *cnrYp* promoter, which could be used by RpoD to mediate a basic expression level of *cnr* as measured in a *∆cnrH* deletion mutant (29) and by CnrH when nickel was present. The promoter motifs had a score of 6 for conservation of the −10 motif that was “TATAAG,” just 2.5 for the −35 motif “CCGAGG” and the penalty for the incorrectly positioned −35 site of 10 yielded an overall score of −1 (Table S4). This was the largest penalty of all sba(RpoD)-associated TSSs upstream of *cnrYp,* indicating that the −35 RpoD element of *cnrYp* was the most “misplaced” −35 site of these promoters. The sequence “GCCG” was identified upstream of the −10 site, so that the overall sequence GCCGTATAAT resembles the ECF291 −10 consensus of CCCTCA (Table 4). The resulting potentially overlapping −10 motif CCGTATA had already been identified in a previous study using primer extension to identify *cnrYp,* and it was verified by run-off transcription (69, 70).

These data explain why *cnr* was shown previously to be expressed at a low level in ∆*cnrH* mutant cells (29), although CnrH was essential for full expression of the determinant (69–71), and why the RpoD promoter motifs were found upstream of *cnrY* (27), but those for an ECF291 promoter were not identified. The −10 sites overlapped, and the frequently occurring RpoD −10 motif masked the rare ECF291 motif, and the positions of the two possible TSSs were so close to each other that they were categorized as resulting from a single promoter. The newly found data in this current study, plus the many previously published ones, support the model of sba promoters as possible hybrid promoters (Fig. 7).

### RpoI and siderophore biosynthesis

RpoI is essential to produce the siderophore alcaligin E in *C. metallidurans* (15, 32, 78, 79). Operon Op0320r_1/2 contains the genes *rpoI, rsiA,* and the genes for siderophore biosynthesis and export. Two promoters were responsible for the expression of this region. A RpoD promoter was upregulated 87-fold in *C. metallidurans* strain AE104 compared to the wild-type, delivering a score of 437, and was associated with TSS_1227516-2 upstream of *acrD* encoding an RND protein, possibly involved in siderophore export (80) and *rpoI*. This promoter is likely under the control of the iron uptake regulator FurA (15). Subsequently, a sba(RpoD) promoter with a distance penalty of 12 and showing a 7-fold upregulation in metal-stressed CH34 cells, yielding a score of 34, was needed for continuation of the transcription of Op0320r_2 of *rsiA,* as well as the downstream genes via TSS_1223590-2 (File S1). The −35 motif of this TSS scored 5.5, the −10 GCGGCTGAAT scored just 4, and the sum of both values was corrected by the penalty of 12 for the incorrectly positioned −35 site. The −10 sequence GCGGCTGAAT contained at the end the motif CTGAAT corresponding to the TATAAT of −10 motifs recognized by RpoD, especially with the strongly conserved final T residue. The −10 consensus-motif of ECF243 promoters is XXCCGGCX with the highly conserved second G residue (Table 4; Fig. S6), which corresponds exactly to the first part of the −10 motif identified by the pattern search, GCGGC. The genes downstream of *rsiA* were not upregulated under iron starvation conditions in a ∆*rpoI* mutant, as shown by RT-PCR (29), indicating that RpoI was essential for expression from this promoter. As indeed was demonstrated to be the case for CnrH and *cnrYp*, a hybrid −10 motif was identified upstream of *rsiA* and annotated as a sba(RpoD) promoter. The new data found in this study, as well as previously published data for RpoI, agree with the sba promoter model (Fig. 7).

### RpoQ and the *gig* genes

The products of the *gigPABT* genes in operon Op1355r_1 play a minor role in copper resistance of *C. metallidurans* (81). They produce a metallophore involved in adaptation to metal stress (82). A *gigT-lacZ* fusion is upregulated by copper ions, especially in cells with disturbed copper homeostasis in the periplasm (81). The genes encoding the sigma factor RpoQ and its membrane-bound anti-sigma factor RsqA are located in operon Op1356f_1, just upstream of the *gig* operon, but on the opposite DNA strand. It was considered that expression of the *gig* operon might be regulated in response to periplasmic copper ions via RsqA and RpoQ; however, this could not be demonstrated.

In agreement with previous data (29), upregulation of *rpoQ-rsqA* depends on the presence of RpoQ (Fig. 5A). The TSS_1315237 + 3 is located 24 bp upstream of *rpoQ*; it is upregulated 9-fold in unchallenged AE104 cells when compared with CH34 cells and has an abundance score of 44. Expression was associated with a PosFail(ECF02) promoter, indicating that the −10 and −35 motifs had a stronger relationship to ECF02 motifs compared to RpoD motifs; however, neither motif was located at the expected position. The conservation score for both motifs with respect to ECF02 promoters was only 6, and the position penalty was 9. The pattern search program identified a GAATAACGGA sequence at the −10 site, which scored only for the A at position 4 of the underlined region. If used, this −10-element would have the final G residue, which should be located at position −7, located at position −3, and therefore, this would be too close to the TSS. A motif related to XXGGTTAC for ECF16 promoters was not found in the region around 10 bps upstream of TSS_1315237 + 3. In this case, the actual promoter elements for *rpoQ* could not be identified because they were likely masked under false-positive and misplaced RpoD motifs.

In contrast, TSS_2431186-3 of the *cop_2_* operon (*copA_2_B_2_C_2_D_2_*) was annotated as an ECF16-controlled promoter, with correctly spaced −10 and −35 sites. The TSS was identified, and transcription was upregulated 487-fold in metal-stressed AE104 cells to an abundance of 2,435. The predicted promoter had a −10 motif of CATTAGGTGG and a −35 motif corresponding to CATTAGGTGG. The −35-motif matched that of ECF16 AATTCG, but the −10 element (GGTTAC) showed only weak similarity to the ECF16 −10 motif. This means that an RpoQ-RNAP would bind to the promoter using the −35 site but should not be able to initiate transcription because the motif at the −10 element lacks conservation. TSS_1328724-3, which controls *gig* operon expression, was annotated as an RpoH promoter, and its promoter elements have no similarity to ECF16 motifs. This TSS is far upstream (13,699 bp) of *gigP* and transcribes also the *rpoQ*-AST_1316290-3, which might interfere with expression of *rpoQ*.

This means that the actual promoter upstream of *rpoQ* was not identified by the pattern search because these motifs were too rare to become readily identifiable against the background of the highly abundant RpoD motifs. On the other hand, an ECF16 motif could be identified due to a strong match to the −35 ECF16 motif in the case of the *copA_2_* operon, but again, only a weak −10 ECF16 motif was present, which matched the effect that RpoQ seemed to downregulate this promoter (Fig. 4). ECF16 motifs also were not found upstream of the *gig* operon, which explains the failure to demonstrate experimentally the requirement of RpoQ for *gig* expression. Instead, the *gig* promoter seems to be far upstream of this operon, and it initiates transcription of a *rpoQ* antisense RNA using RpoH. This makes sense, since cytoplasmic Cu(I) ions should bind to thiol groups and denature them, leading to upregulation of the RpoH regulon, subsequent expression of the metallophore-producing *gig* determinant and via the AST downregulation of *rpoQ* expression. This has the consequence of upregulating expression of the gene encoding the periplasmic Cu(I) oxidase, CopA_2_, to decrease import of copper into the cytoplasm (81–83).

### 6S RNA, RpoD, and RpoS

Transcription of the *ssrS* gene encoding the 6S RNA was initiated from TSS_3038511 + 2, which was upregulated 14-fold in metal-stressed cells to an abundance score of 81,591 (Fig. 3; File S1). In *E. coli*, the 6S RNA inhibits specifically RpoD-dependent promoters with a weak −35 site, or when they have an extended −10 (84). In the latter case, the presence of a strongly conserved −35 element overrides this inhibition (85, 86). Sigma region 4.2, which binds the −35 element during transcription initiation, is critical for the interaction with 6S allowing it to compete with the promoter for binding by sigma 4.2 (85, 86). In *E. coli*, the cellular concentration of 6S increases in the stationary phase (87). Transcription of 6S is inhibited by some DNA-binding proteins such as H-NS and LRP, with FIS having a dual regulatory role (87).

The 6S RNA is present in many bacteria (61) and may have roles other than that in *E. coli*. In general, 6S is a guardian regulating the economic use of cellular resources under limiting conditions and during stress. The 6S RNA interacts only modestly with other RNAP holoenzymes, core RNAP, or unbound sigma factors, although altered expression was not strictly confined to RpoD promoters but included many genes under the control of alternative sigma factors (87).

In particular, RpoS-dependent promoters can be affected by the 6S RNA, although this is still debated (84). Due to the similarities between the −35 elements recognized by RpoD and RpoS (TTGACA vs. GCTACAA (49)), the 4.2-domains of both sigma factors should be related so that it can be expected that both 4.2 regions are able to interact with the 6S RNA, although perhaps with a lower affinity in case of RpoS. In *C. metallidurans*, there is a large group of promoters that shows partial RpoD- and RpoS-dependence. RpoS is considered to be the central regulator of the general stress response in bacteria, which answers specifically to nutrient deprivation (49). Many bacteria live under starvation conditions in their native environments and are unable to grow at their optimal growth rate. Consequently, RpoD and RpoS likely cooperate to adjust the transcriptome to the respective starvation conditions the bacterium is actually facing, so that RpoDS hybrid promoters may well be the consequence of the fact that bacteria are always starving under natural conditions, sometimes more, sometimes less.

Promoters of the sba(RpoDS) type would have been identified in the past as promoters with a weak −35 element because the real −35 element would be up- or down-stream of the expected position, which is about 16 bp upstream from the −10 site. Considering the 6S site as guardian to regulate the economic use of cellular resources, plus a scenario with a constant transition between more RpoD- or more RpoS-usage, this would indeed link the effect of these two sigma factors with that of the 6S RNA. In *E. coli*, the majority of RpoD-RNAP could be sequestered by the 6S RNA (85), which would leave sba promoters open for transcription initiation by RNAP holoenzymes using alternative sigma factors (Fig. 7).

Expression of *ssrS* in *C. metallidurans* seems to be a complex process. The responsible promoter upstream of the TSS_3038511 + 2 was annotated as a sba(ECF243) promoter, assigning this promoter to the iron-specific ECF sigma factors RpoI, RpoJ, or RpoK. Although the region directly downstream of this TSS is indeed increased in its abundance in metal-stressed *C. metallidurans* cells (Fig. 3, middle panel compared to the other ones), the 6S abundance is not changed (Fig. 3; Fig. S7), and as in other bacteria, the 6S RNA is not essential in *C. metallidurans* (Fig. S8) (88). In gene arrays (89), region NC_007973.inter.2408 between Rmet_2791 (*zapA*) and Rmet_2792 (Fig. 3) is not changed in expression in any of the gene array experiments nor is synthesis of the corresponding two open reading frames. The region is in the vicinity of the *btu/cob* cluster for cobalamin uptake and synthesis depends on cobalt, which is associated with ASTs in a complex pattern. One AST antagonizes *ssrS* and proceeds into the *btu/cob* cluster. It is transcribed by a sba(RpoD) promoter and is upregulated 10-fold by metal stress in AE104 cells. This would mean that expression of *ssrS* in *C. metallidurans* would be under the control of iron availability and metal stress via interference of an antisense RNA and the transcript initiated from TSS_3038211 + 2.

### Function of sba promoters in *C. metallidurans*

Nearly half of the TSSs in *C. metallidurans* could be assigned to sba(RpoD) or sba(RpoDS) promoters, the latter used by either sigma factor in response to the actual nutrient supply or limitation conditions. For this type of promoter, the −35 element is too close or too far away from the −10 element to allow efficient positioning of the RNAP at the −10 site, so that Up-like elements, activators, the extended −10 site, or even the upper part of the discriminator becomes responsible for RNAP positioning at the −10 site. Additionally, RNAP holoenzymes with other sigma factors bound may recognize the respective promoter by using their own −35 element, which overlaps with the respective RpoD motif or is in the vicinity. This would not only lead to competition between the sigma factors for the RNAP core complex but also to competition between the resulting RNAP holoenzymes for the sba promoters, leading to the inhibition of gene expression as shown in some cases here. When RpoH, FliA, or an ECF sigma factors are removed from competition by degradation, binding to a soluble, or to a membrane-bound anti-sigma factor, respectively; bound RpoD- or RpoS-RNAP would be allowed to slide to the −10 element in a process assisted by an UP element, extended −10 or discriminator, leading to transcription initiation on a low frequency level. This would guarantee expression of the respective operon on a low constitutive level even in the absence of the alternative sigma factor, for instance, triggered by a mutation or gene deletion. In the case of imbalanced nutrient supply or stress, the 6S RNA may sequester RpoD-RNAP so that the sba promoter is open for other RNAP holoenzymes, which become available when specific signals release such a sigma factor from its anti-sigma factor or intervene with its degradation. In this way, sba promoters are effectively tools to break the dominance of RpoD as the central factor of transcription initiation in times of stress or nutrient deprivation (Fig. 7). Although RpoD is the one sigma factor that rules them all, sba promoters limit the consequences.

## MATERIALS AND METHODS

### Standard methods

*C. metallidurans* wild-type strain CH34 and its plasmid-free mutant AE104 (4) were used in this study. Growth conditions were used exactly as published (4), and the improved ToxMix (multi-metal toxicity mixture) to challenge the cells has been reported recently (26, 28). To measure the activity of promoter fragments, they were cloned upstream of a promoter-less *lacZ* gene on plasmid pVDZ’2 and transferred into *C. metallidurans* cells by conjugation to measure specific activity of the reporter gene product, β-galactosidase (54, 90–92). RNA isolation and (qRT)-PCR methods were also carried out exactly as reported (26). Bacterial strains and plasmids are presented in Table S5, and the oligonucleotide primers are available upon request.

### Determination of the TSSs

Cells of *C. metallidurans* CH34 wild type and its plasmid-free derivative, strain AE104, were cultivated in Tris-buffered mineral salts medium with gluconate as the carbon source to the mid-exponential phase of growth (120 Klett units) and challenged for 10 min with 3.35 mM MultiTox metal mix for strain CH34, 1 mM MultiTox metal mix for strain AE104, and with 50 µM EDTA for either strain, or cells remained unchallenged, as published (26). From each of the three independent cultivations, RNA was isolated and used to determine the published transcriptome (26), and the TSSs using the Cappable-seq enrichment strategy (53). The quality score was calculated from the relative read scores for each TSS, which were named as “TSS_” followed by the position of this TSS, “+” or “-“ for the DNA strand, and “2,” “3,” “4,” and “5” for the chromosome, chromid, plasmid pMOL30, or pMOL28, respectively, as done previously (27). Since the TSS quality score defined the enrichment ratio of 5’-triphosporylated RNA divided by depleted 5′ positions stemming from RNA degradation or processing, the ratio of the quality scores of a TSS found in cells that were cultivated under different conditions indicates a change in the abundance of the respective 5′-triphosporylated RNA, meaning an up- or down-regulation of initiation of transcription at this specific position.

The newly identified TSSs in the six data sets were compared with each other and the 5,763 TSS signals obtained for non-challenged *C. metallidurans* wild-type cells in the published previous experiment (27). Since signals only appearing in all three biological repeats had been used in the previous version, 1,189 TSSs that appeared only twice were added to the 5,763 TSS signals, totaling 6,952 TSS signals in the published basic data set for unchallenged CH34 cells. In the comparison of the total of seven data sets, TSSs were counted as one when they were on the same replicon and DNA strand, and within a distance of 5 bases of each other. In most cases, the distance was 0 or 1 base position separation. Binning of the TSSs into two databases was done in a stepwise manner. First, 4,229 TSSs from unchallenged CH34 cells (C0) that could not be assigned to TSSs in the published database (6,952 TSS signals) were identified as newly identified TSSs. Second, 2,223 TSSs, which were new and appeared in metal-challenged CH34 cells (CM), along with the 666 TSSs that were new in EDTA-challenged CH34 cells (CE), plus the 657 new TSSs in unchallenged AE104 cells (A0), 724 in metal-challenged AE104 cells (AM), and finally the 308 TSSs in EDTA-challenged AE104 cells (AE), totaling 15,758 TSSs that appeared in at least one of the seven data sets, were then binned. Some of the TSSs have been previously published (26, 28, 54), but not analyzed in detail, and not in comparison with the global TSSs, nor had they been assigned based on metal-challenged or metal-starved cells of strains CH34 and AE104.

When the quality scores of individual TSSs in unchallenged CH34 cells in the published (27) and new version were compared (Fig. S1), the new scores were roughly six times higher than the published ones, probably due to an increased efficiency of the Cappable-seq enrichment protocol. This prevented calculation of the score ratios of the previously published and newly identified TSSs in unchallenged cells of strain CH34. TSSs from the published data set not found in the new one were omitted from further comparisons. Since division by 0 is not possible during these comparisons, a score of 5 and a deviation of 10 was attached when a TSS was not found in a strain and under a condition tested. A TSS score of 60 in the new determination would equate to the cutoff of 10 in the published analysis. In broader terms, a TSS score of 50 and below would have some probability of being a false-positive signal. Such TSSs may have originated from degraded RNAs, although the Cappable-seq enrichment protocol should prevent detection of such RNAs (53). TSSs with scores above a threshold of 50 should be meaningful, including those between the technical cutoff of 10 and 50, with a probability increasing with the score.

In the published data set, the mean ratio of the log_10_ values of the abundances of the gene transcripts (NPKM values, nucleotide activities per kilobase of exon model per million mapped reads), and the TSS scores was 0.94 ± 0.48 (27). This indicated that the promoter associated with a TSS could be responsible for a gene transcript if the TSS score was between 0.33-fold and 3-fold of the respective NPKM value of the subsequent gene. Since the NPKM values for the published and the new data sets were in the same range (26, 29), but the scores of the TSSs in the new data were set 6-fold higher, a promoter responsible for a gene transcript should deliver a TSS with a score twice or 18-fold higher than the NPKM value of the affected downstream gene. Including a wider margin, the threshold for a promoter should have a TSS score > the NPKM value. This defined quality criterion for a TSS with respect to its impact on the transcriptome indicates that the TSS score should be larger than 50 or the NPKM value. Third, a promoter responsible for a regulated gene should initiate a TSS regulated under the same conditions and the gene transcript.

After comparison of the abundance scores between the TSSs of the different cells and to facilitate the subsequent construction of the transcriptional landscape of *C. metallidurans,* a regulation tag was associated with a TSS. It shows “NONE” when all changes of the scores were < 2-fold and > 0.5-fold. Otherwise, it states “REG” followed by the rounded ratio of the largest and smallest scores, and after “@” the condition of the highest score, “C” for CH34, “A” for AE104, “M” metals, and “E” for EDTA or “0” for control cells (Calculation in TSS_Regulation.xlsx, summary in File S3 in the Supplement). TSSs with a regulatory tag of “Reg_3” or higher numbers were considered regulated TSSs.

### Transcriptional landscape

To construct a transcriptional landscape of metal-stressed *C. metallidurans* cells, first, the gene regions affected by an antisense element (AST) in the published data set (26) were annotated to the corresponding AST element based on the impact of this element. Subsequently, regulation tags were also attached for ASTs, genes (NPKMs), and 5′ and 3′ untranslated regions (5 UTRs and 3 UTRs, respectively). Finally, these elements were associated with the respective TSS upstream, which defined the impact of a particular TSS.

While the positions of all sense transcripts were defined by that of the annotated open reading frame or RNA gene, ASTs could be one transcript or a bin of transcripts opposite to sense elements. In the latter case, the 5′ end of an AST may vary with the growth condition of the cells with respect to metal availability (26). Consequently, TSSs were associated to ASTs when they were located upstream or even within this antisense element. Such TSSs could be responsible for just a part of an AST bin. TSSs associated to sense elements were always associated with a gene, for example, in the case of protein-encoding genes an open reading frame. Since the 5′ ends of asRNAs may vary from condition to condition; however, for sense and antisense-associated TSSs, the distance to the closest gene was always indicated (in the data set as locus tag of an individual TSS as “Rmet_xxxx:distance”). This resulted in a global transcriptional landscape (File S1 in the Supplement), which summarizes for each element its regulation in response to changes in metal availability. The impact of a TSS was the sense (5UTR, NPKM, and 3UTR) or antisense (AST) element downstream of the TSS in the direction of the respective DNA strand of the chromosome, chromid, or plasmid, which of an AST the affected sense elements, which of an 5UTR or 3UTR the gene downstream or upstream of it, and in the case of a gene (NPKM), the name of the gene, Rmet-number, predicted gene product, its association to an operon, and the function of the gene product as defined by the KEGG orthology system (93, 94).

### Finding and scoring promoter consensus motifs

Possible promoter consensus motifs appearing between −90 and + 10 of the TSSs were searched using the published discovery software and scoring algorithm for the degree of conservation of the consensus sequences (27). Subsequently, the motif scores of each TSS were corrected for the correct position of the −10 and −35 sites. No penalty was added when the −10 sites started at positions −13 or −12, a penalty of −0.5 at −14 or −11. Other positions yielded a penalty which was the distance to position −12.5. Similarly, the penalty for the position of the −35 site was zero for positions -37,-36 or −35. Otherwise, the penalty was the distance to position −35.5. The motif score minus the sum of both position penalties gave the final score of the promoter consensus motifs.

The promoters were subsequently grouped into four categories. In the case of “PosFail” (position failed) promoters, the −10 and −35 consensus motifs were outside of the correct positions; in the case of “ba” (blocking, another sigma factor), the −35 site was at the correct position but the −10 site was not. In the case of “sba” (sliding, blocking, another sigma factor), the −10 motif was at the correct position, but the −35 site was too far upstream or downstream of the correct position (Fig. 2). If both motifs were at the correct position, none of the comments was added.

In the next step, the consensus motifs were scored. Discrimination between the motifs for RpoD and RpoS were scored in two steps. First, differences between the extended –10, –10, and discriminator consensus motifs (48, 49) were used to assign the upstream sequences of the TSSs to RpoD, RpoS or both, and RpoDS. In a second step, for all sigma factors, consensus motifs were generated. The ECF sigma factors were newly categorized (Fig. S5), the consensus motifs for the respective ECF groups were taken from the ECF database (56), RpoS, RpoH, and FliA were taken from other sources (48, 57, 58), while RpoN was not included. All these data were used to generate a scoring system (Table S; Table 4). In this way, all TSSs were annotated to sigma factors, and the groups sba, ba, PosFail, or Position OK.

### Statistics

Students’ *t*-test was used, but in most cases, the distance (D) value has been used several times previously for such analyses (89, 95, 96). It is a simple, more useful value than Student’s *t*-test because non-intersecting deviation bars of two values (D > 1) for three repeats always means a statistically significant (≥ 95%) difference provided the deviations are within a similar range. At *n* = 4, the significance is ≥ 97.5%, at *n* = 5 ≥ 99% (significance), and at *n* = 8 ≥ 99.9% (highly significant).

## Data Availability

RNASeq data were deposited under BioProject PRJNA753702.

## SUPPLEMENTAL MATERIAL

File S1 jb.00219-26-s0001.xlsx Transcriptional landscape of the four replicons and the changes under conditions of metal availability.

File S2 jb.00219-26-s0002.xlsx Regions encoding genes involved in metal homeostasis extracted from the transcriptional landscape.

File S3 jb.00219-26-s0003.xlsx List of all TSSs with their abundance, regulation, impact, and assignment to sigma factors.

File S4 jb.00219-26-s0004.xlsx All TSSs and their regulation of abundance listed by the replicons and DNA strands.

File S5 jb.00219-26-s0005.xlsx 10 TSSs with the highest score assigned to sigma factors.

Supplemental material jb.00219-26-s0006.pdf Supplemental results, Figures S1 to S8, and Tables S1 to S5.

ASM does not own the copyrights to Supplemental Material that may be linked to, or accessed through, an article. The authors have granted ASM a non-exclusive, world-wide license to publish the Supplemental Material files. Please contact the corresponding author directly for reuse.
