# Supplementary material for: Transcriptional landscape of metal-resistant Cupriavidus metallidurans: one sigma factor to rule them all
Source: J Bacteriol. 2026 Jul 27;208(8):e00219-26. doi: 10.1128/jb.00219-26 (PMC13492112; doi:10.1128/jb.00219-26)
Supplement: Supplementary file 1 [file jb.00219-26-s0006.pdf]

Supplement

Contents

List of additionally excel data files 2
Extended Results 2
Dominance of RpoD/S-depending promoters *2*
Motifs recognized by other sigma factors *3*
Metal homeostasis genes *4*
Supplementary Figure S1. The new TSS determination reached higher TSS quality scores compared to the published one. 6
Supplementary Figure S2. Multiple alignment of the 11 ECF sigma factors from *C. metallidurans* 7
Supplementary Figure S3. Alignment of RpoE orthologs from *C. metallidurans* and *E. coli*. 8
Supplementary Figure S4. Alignment the ECF sigma factors from *C. metallidurans* with 95 other ECF sigma factors. 9
Supplementary Figure S5. Reclassification of the sigma factors from *C. metallidurans*. 10
Supplementary Figure S6. Predicted consensus sequences for groups of ECF sigma factors. 11
Supplementary Figure S7. Expression of *ssrS* in *C. metallidurans*. 12
Supplementary Figure S8. Growth of C. metallidurans strains CH34 wild type and AE104 in comparison with their ΔssrS deletion mutants. 13
Supplementary Table S1. Scores for the bases in the promoters of sigma factors 14
Supplementary Table S2. Testing the scores for the promoter motifs against each other 15
Supplementary Table S3. Table Overview Promoters including the second-best consensus motifs for sba and ba promoters 16
Supplementary Table S4. Transcriptional landscape of the cnr resistance determinant on plasmid pMOL28 20
Supplementary Table S5. Bacterial strains and plasmids used 21
References of the Supplement 22

List of additionally excel data files

1. TSS_Regulation.xlsx: For each replicon and DNA strand, the TSSs are listed plus the abundance (mean value, deviation) under all three conditions and for both strains (CH34, AE104). The abundances were compared (ratio Q for each comparison plus D value, the distance of both data points divided by the sum of the deviations) and the comparison used to derive the regulatory tag for each TSS.
2. TopTen.xlsx lists the ten TSSs per sigma factor by their abundance.
3. TSS_Summary.xlsx: All TSSs are listed with their maximum abundance, promoter assignment, second best -10 or -35 site and the impact on genes and ASTs.
4. Landscape.xlsx: For each replicon and DNA strand, the genes (NPKM), 5UTRs, 3UTRs, antisense RNAs (AST) and TSSs are listed with their maximum abundance, the regulatory tag, operon (genes), or sigma factor-assignment for genes in the KEGG orthology category. The impact lists for each item either the function of the gene product (genes, 5UTR, 3UTR), for ASTs the affected genes and for the TSSs the affected genes and ASTs. This provides a complete inventory of the TSSs in metal-challenged or metal-starved CH34 and AE104 cells, which sigma factor is responsible for a TSS and which sense or antisense RNA is initiated at a respective TSS.
5. Regions.xlsx is an extract from the landscape and assigns selected gene regions to sigma factor genes, genes encoding proteins involved in metal resistance that act in the periplasm, polyphosphate, glutathione, uptake or efflux across the inner membrane, or regulators involved in metal homeostasis.

Extended Results

Dominance of RpoD/S-depending promoters. For all promoters, the highest-scoring promoter driving expression of the gene encoding the cold-shock protein CspA had an abundance score of 238,493 and was categorized as “ba(RpoD)”. This promoter had a score for the RpoD motifs of 14 but since the -10 and the -35 sites were only 11 bp apart from each other and not 16 as optimal, and the -10 site had an A at position -10 instead of the highly conserved T at position -7 (1), which did not agree with the high abundance of the TSS. A possible explanation could be a very stable 5’end of the *cspA* mRNA, or some binning artifacts. This promoter might hide an interesting regulatory process.

The mean maximum abundance scores of the strongest TSSs were 146,722±39,622 and most of them were categorized as RpoD/S or sba(RpoD/S). Promoter number 10 was the RpoD-dependent promoter *zntAp* in front of the gene encoding the main zinc-exporting P-type

ATPase ZntA, which was fully up-regulated in metal-stressed cells with an abundance > 100,000 (Suppl. Table TopTen.xlsx). The mean maximum abundance scores for RpoD/S and sba(RpoD/S) promoters were similar with 105,539 ± 43,536 and 105,520 ± 22,905, respectively, so that these sba promoters were not different in their activity compared to those with a correctly positioned -35 site. The clearly RpoD- or RpoDS-dependent TSSs were led by the 5' end of the mRNA encoding Rmet_2142, which is a predicted cell wall-associated hydrolase, along with that encoding the small protein Rmet_5969 with unknown function encoded adjacent to the *czc* region and strongly up-regulated under metal stress, the gene encoding a pilus protein, *zntAp*, *chrA*$_1$*p,* which controls chromate efflux, and the promoter of the gene encoding the transcription termination factor, Rho. The sba(RpoD) and sba(RpoDS) promoters were associated with genes encoding several proteins of unknown function, but also those encoding the phosphate sensor, PstS, enzymes for the synthesis of branched chain amino acids, the RNA chaperone Hfq and the main regulator of iron uptake, FurA.

Not including *cspAp*, the ba promoters for RpoD and RpoDS were lower in maximum abundance than the sba promoters with 22,003 ± 13,657 of the nine most abundant TSSs, the respective ten PosFail promoters with 17,381 ± 11,642. In addition to *cspAp*, ba(RpoD) or ba(RpoDS) promoters were found upstream of *clpS* encoding a protease adapter protein, *dnaA* encoding the initiator of DNA replication and *ihfA* encoding one subunit of the integration host factor. A PosFail(RpoDS) promoter ranking at position 13 was 91 bp upstream of *rpoH*, which encodes the heat-shock sigma factor. This promoter might be in fact RpoH-dependent. This indicated that ba and PosFail promoters might also lead to TSSs with high abundance, that they initiate transcription of important genes, but that they may also mask promoters used by other sigma factors. In comparison, the ten RpoS-dependent promoters with correctly positioned -35 sites had an abundance of only 1,980 ± 884, which is 10% of the level of the corresponding sba promoters.

<u>Motifs recognized by other sigma factors</u>. The dominance of sba-type promoters opened up the possibility that a -10 region might indeed contain overlapping motifs for two sigma factors, resulting in two TSSs that were fused during the binning into one. *C. metallidurans* contains in addition to RpoD/S and RpoN, the latter of which was not addressed here, a heat-shock sigma factor RpoH, a motility sigma factor FliA and 11 sigma factors of the extracytoplasmic functions (ECF) family.

These ECF sigma factors are mainly composed of the sigma factor domain 2, which binds to the -10 promoter motif, and domain 4.2 binding to the -35 site (2, 3). Both domains are connected by a linker region of about 40 amino acyl residues, that exhibited a low degree of conservation and was specific for the respective sigma factor (Suppl. Fig. S2). With this, the

11 ECF sigma factors were not different from RpoE from *E. coli* (Suppl. Fig. S3) or most ECF sigma factors that were randomly taken from the data base (Suppl. Fig. S4).

The numbers of predicted promoters (Table 3) were between 395 for family ECF291 (CnrH) and 52 for ECF18 (RpoP), with 211 assigned to the negative-control group ECF11, which, however, belongs to ECFs with similar consensus sequences. Nevertheless, the number of 211 TSSs assigned to an ECF group with no representative in *C. metallidurans* indicated that when only a small number of about 200 TSSs per group were found, this result may represent a false-positive and should be considered with caution. This restriction applied to FliA and all ECFs except ECF291.

As in the case of RpoD, the number of sba promoters was higher than that of those with correctly positioned -35 and -10 sites. For all ECF sigma factors and FliA, the number of PosFail promoters was higher than that of the sba and correctly positioned motifs (Table 3). Because ECFs were not able to use an extended -10 or a discriminator for correct positioning of the RNAP at the -10 site, sba, ba, and PosFail promoters assigned to an ECF group had a lower probability of being a real promoter compared to the RpoD promoters.

The TSSs with the highest abundance scores for alternative sigma factors were also listed (Suppl. Material TopTen). Control of expression of *clpB* (4) by RpoH and of *flhD1* for a flagellar transcriptional activator by FliA agreed to the physiological functions of these sigma factors. On the other hand, and in agreement with their assignment as result to be taken with caution, not much insight came from the analysis of the promoters for the ECF sigma factors. The ECF291 promoter, with the highest score of 2,751, was upstream of an *mmf* operon encoding largely uncharacterized gene products.

Metal homeostasis genes. Using an intermediary data file (Supplement Regions.xlsx), the promoters associated with the TSSs upstream of genes encoding gene products involved in metal homeostasis were extracted from the transcriptional landscape data (Supplement Landscape.xlsx). Moreover, the second-best sigma factor able to bind the respective -10 sequence or -35 sequence were also obtained (Suppl. Table S3). The TSSs for most metal homeostasis genes were under the control of RpoD or RpoS (Table 5).

The promoters for genes of inner membrane uptake systems for metal cations or phosphate were under the control of RpoD (*zupT, pitA, feo*) or RpoS (*mgtA*) with correctly positioned elements, or sba-type promoters assigned to the same sigma factors (*$corA_1$, $corA_3$, zntB, pstSCABU*). The RpoS-assignment of *mgtAp* was in agreement with its activation of the RpoS-dependent stress systems caused by magnesium starvation (5), which, for *mgtBp*, was via a ba(RpoDS) promoter. Sigma factors ECF207 and ECF291 may contribute towards transcription initiation events for these genes with low activity. Genes encoding proteins

involved in phosphate, iron and zinc storage were under sba(RpoD), sba(RpoDS), RpoD or RpoS control, as were most regulators for metal and phosphate homeostasis (Table 5).

The genes for P_{IB}-type ATPases involved in export of divalent transition metal cations, along with *cdfX* encoding a CDF protein and $chrA_1$ encoding the plasmid-borne chromate efflux pump, were all under RpoD control, those encoding copper-exporting enzymes, together with the genes encoding the three remaining CDF proteins, as well as those encoding $ChrA_2$, ArsB and AtmA are under the control of sba(RpoD). In the case of the genes encoding the two P_{IB}-type enzymes that export copper into the periplasm for anabolic processes, the promoter for one was categorized as sba(ECF291), while the other belonged to the sba(RpoD) grouping. Again, other sigma factors may contribute to mediate weak transcription initiation events. In this case, RpoH possibly controls expression of *fieF*, ECF291 controls *atmA* expression, and other sigma factors presumably control the expression of the corresponding antisense RNAs. The genes encoding trans-envelope efflux complexes and metal homeostasis factors acting in the periplasm were also mainly under the control of sba(RpoD/S) or RpoD, with a small contribution made by sba-promoters assigned to other sigma factors (Table 5).

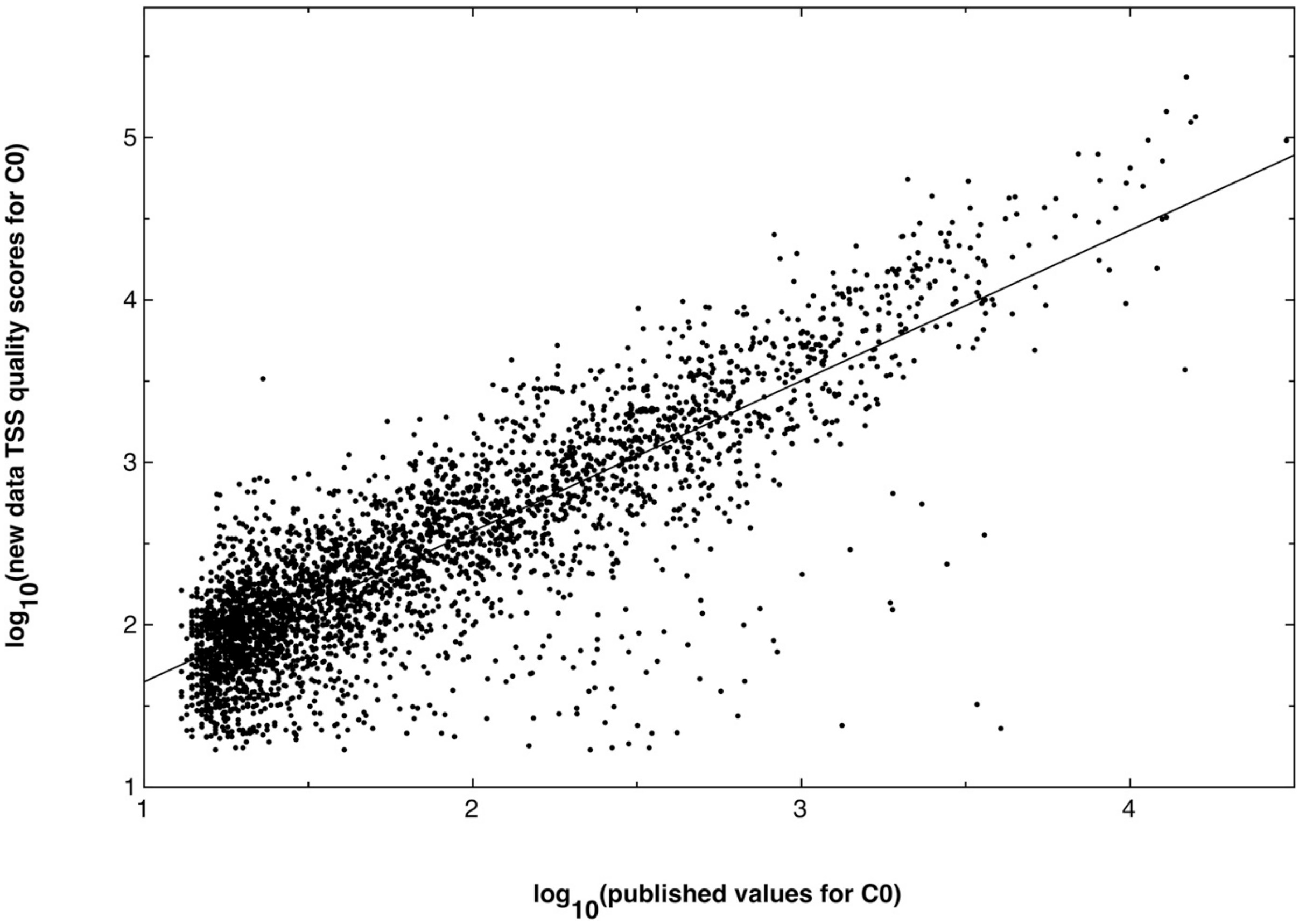

Supplementary Figure S1. The new TSS determination reached higher TSS quality scores compared to the published one.

The figure shows a logarithmic plot of the quality score of TSSs determined in non-challenged *C. metallidurans* CH34 cells in the new measurement compared to the published one (6). The indicated result of the linear regression (correlation coefficient 85.1%) was $\log_{10}$(new) = 0.7726±0.0191 + 0.9265±0.0097 * $\log_{10}$(published), which is roughly $\log_{10}$(new) = 0.77 + $\log_{10}$(published), equaling new value = 6-times the published value.

```
Consensus       MA---------------AGDRRXXX------XLASRH------XALXGLARRLLXBXXDA   33
ABF07483, RpoO  MD-------A-------ANTRRRFD------DLVLPH-----LDAAYNLARWLCGNPGDA   35
ABF07796, RpoR  M----------------ATDQELSD------FLASVE------RRAFKQAVFAVRDDEAA   32
ABF08006, RpoI  MA---------------STRLRALG-------LYVHY------QELVGTWTRRLSNRHEA   32
ABF08531, RpoP  MA--LSPEPPSSLPDAPTGLDVLLGRVALGDRLALRALYDQVVPKLFGLALRITRRRDWA   58
ABF09302, RpoE  MS--EREADQLLVERVQQGDKRAFE------LLVTKY-----HRKIIRLISRLVRDPAEV   47
ABF10152, RpoL  ME---------------PFERQLV-------VLA---------PRLRRHARGLTGDAMLA   29
ABF10869, RpoK  MA-------------ADAGPHHDIQ------ALYSCH-----HRWLLGWLHRKIGCWHRS   36
ABF11364, RpoJ  MD---------------PGTRSQVD------VLYSRH-----HAWLQGWLLRKIGNACDA   34
ABF11548, RpoQ  MGALTYHFEAITPEPRDDVDRSNLRAVVACMKPAPERAGMEAMDAVEQRLRALLLAGLGG   60
ABF12259, RpoM  M-------------------------------I----------PALRRYARALLREQAAA   19
ABF13066, CnrH  MN--PEDADRILAAQAASGNQRAFG------QLVARH-----GVALAQAARSFGIPETDV   47

Consensus       DDXVQDAFXRAL--RARXDFR--IAZPRAWLXXIAXNTAJ------DTLRRR--------   75
ABF07483, RpoO  DDVVQEAFLRAF--RLFGSFR--GDQPRPWLLAIVRNTWF------TTWQKRKAAA--E-   82
ABF07796, RpoR  LDIVQDAMIKLA--EKYGDKS--AAELPPLFQRILQNTIH------DWFRRQKVRNTWVT   82
ABF08006, RpoI  EDLSQEAMLRVL--EARVT----PEQPRAYLHQTARNIAI------DTFR----------   70
ABF08531, RpoP  EDVVQESFVSIW--HHAADYRPQVAAPMTWMTTIVRNRAL------DCLRRAHVA-----  105
ABF09302, RpoE  EDVAQDAFIKAY--RALPQFR-GESAFYTWLYRIAVNTAK------NYLATQGRRP---E   95
ABF10152, RpoL  DDLVQDTLERAL--RYRWRFR---LRPGALWGDGADGLLP------WLLTLMHRL-----   73
ABF10869, RpoK  ADLAHDTFMRLL--AREEALA--IAEPRAFLTTVAQRVLA------NQWRRE--------   78
ABF11364, RpoJ  ADIAQDVFVRVL--HRKVVE---IREPRTYLGTIANGLVI------DFWRRR--------   75
ABF11548, RpoQ  DQAAYHAFLTELTGRLRAFVRRRLYQLQDEVEDIVQETLLAVHNARHTYRAEEPLTAWVY  120
ABF12259, RpoM  DDLVQDCLEKVIT---YWDSRRNRADTRSWVFAILHNLAM------TALRRH--------   62
ABF13066, CnrH  DDVVQDTFVAAW--HALDDFDP-DRPFRAWLFRIGLNKMR------DLYRFRRVRQ----   94

Consensus       ---------XLEQAELLDLXEAPDALAXXXSPEEDLHARQXLLXXXXALXRLPXXQREXF  126
ABF07483, RpoO  ----------VEYDEAAPPGELPDWHGGPGDPEQWLVRDEDVRLVHAALERLPVVFREVL  132
ABF07796, RpoR  LFSNLRDEREGDDSDLLDTLEAQAGTEMAESSADKVERAQVMHIIEQEIQRLPTRQREAF  142
ABF08006, RpoI  ---------KRDGHEHLPLDSIESIHAPSGNPEADLHAVQVVAGLERALAELPLKCRQVF  121
ABF08531, RpoP  ---------RLASATSLEDTPEADLEDEHDGPVELAQASEQARALNRCLQRLDGQQREAV  156
ABF09302, RpoE  A-------SSDIDAEEAETFADGEQLRDINTPESMLHTRQVADTVNRAMEALPEELRTAI  148
ABF10152, RpoL  ---------RLNHLRKSDVVTAMDTLPEVVAHTADLGLRRDLL---QALGQLPEPQRAVL  121
ABF10869, RpoK  ---------KLEQAYLEALASRPAALAP--SEEERAILLETLNDIAERLDGLPVVVKRAF  127
ABF11364, RpoJ  ---------ELEQAWLETLAQLPEAHTP--SPESRLHFLQTLIEIDQMLDTLKPNARTAF  124
ABF11548, RpoQ  AIARYKLLDFLRSRSRHGATESLDDVADILCHSDDAPAHDARLDLSGLLEQLPDKQRLPI  180
ABF12259, RpoM  ---------ASAQGLHVAIDDVAESLMTVPPAQEDALRYRDLL---SAVDALPAEQRSVL  110
ABF13066, CnrH  --------FLFGAENLGDLELAGGVANDEPGPEQQVAARLELARVASTLGKLDTGSREVI  146

Consensus       LLXXLEGLSYAEIARXLGISVGTVKSRISRAREKLAAXLRAXX-----------------  186
ABF07483, RpoO  VLRELEELHYRDIARIADIPVGTVMSRLARGRRMLAAAVVAVRDEGAVRLAHPDAPNTAN  192
ABF07796, RpoR  LMRYWEDMDVAETAAVMGCSEGSVKTHCSRATHTLAQALRARGVRL--------------  202
ABF08006, RpoI  IWQRLDGCSQQEIAERLNISKNMVEKYMIRAMRHLRDQLGQLSPH---------------  181
ABF08531, RpoP  VLAYLQDLSHAELAARLRVPLGTIKSWIRRGLEKLRACLEATP-----------------  216
ABF09302, RpoE  TLREIEGLSYEEIAEAMGCPIGTVRSRIFRAREAIAEKLRPLLGTAEGKRW---------  208
ABF10152, RpoL  LLVSLEQFSYAEAARVLDVPIGTVMSRLARAREHMRRLLDGGADGAAATTSL--------  173
ABF10869, RpoK  LMAQLEGQSQAEIASALRISVTTVKRYLVRAGTKCYFAMSLA------------------  187
ABF11364, RpoJ  LLAQLDGLTCPQIAERIGMSLATVERYISQALRRCYALQFGA------------------  184
ABF11548, RpoQ  LHVKLEGLSVAETARLTGLSESAVKVGIHRGLKALAAKARLFTS----------------  240
ABF12259, RpoM  LLVSVEEMSYAETARILEIPVGTVMSRLSRAREKLLRQMEPGLKAAPTSGLRR-------  163
ABF13066, CnrH  VLTAIVGMSQPEAAAVLGLSVKAVEGRIGRARAKLSALLDADSEK---------------  206

Consensus       -----------------                                            169
ABF07483, RpoO  APADIVQPAGGTRHGLQ                                            209
ABF07796, RpoR  -----------------                                            188
ABF08006, RpoI  -----------------                                            166
ABF08531, RpoP  -----------------                                            199
ABF09302, RpoE  -----------------                                            199
ABF10152, RpoL  QR-------------VK                                            177
ABF10869, RpoK  -----------------                                            169
ABF11364, RpoJ  -----------------                                            166
ABF11548, RpoQ  -----------------                                            224
ABF12259, RpoM  V---------------K                                            165
ABF13066, CnrH  -----------------                                            191
```

Supplementary Figure S2. Multiple alignment of the 11 ECF sigma factors from *C. metallidurans*

These ECF sigma factors are mostly composed of sigma factor domain 2 that binds the -10 region (yellow box), 4.2 binding to the -35 motif (green box) and a linker region in-between.

```
Consensus       MEIWFGETLPRMSEXXXDQXLVERVQXGDXXAFXLLVXXYXXKXXXLXSRXVXXXXXVXD   60
ABF09302, RpoE  -----------MSEREADQLLVERVQQGDKRAFELLVTKYHRKIIRLISRLVRDPAEVED   49
BAA10920        MEIWFGETLPRMSEQLTDQVLVERVQKGDQKAFNLLVVRYQHKVASLVSRYV-PSGDVPD   59

Consensus       VXQXAFIKAYRALXXFRGXSAFYTWLYRIAVNTAKNYLXXQGRRPXXSSDXDAXEAEXFX  120
ABF09302, RpoE  VAQDAFIKAYRALPQFRGESAFYTWLYRIAVNTAKNYLATQGRRPEASSDIDAEEAETFA  109
BAA10920        VVQEAFIKAYRALDSFRGDSAFYTWLYRIAVNTAKNYLVAQGRRP-PSSDVDAIEAENFE  118

Consensus       XGXXLXXIXXPEXXXXXXZXXXXVXRXXEXLPEXLRXAITLREJXGLSYEEIAXXMXCPX  180
ABF09302, RpoE  DGEQLRDINTPESMLHTRQVADTVNRAMEALPEELRTAITLREIEGLSYEEIAEAMGCPI  169
BAA10920        SGGALKEISNPENLMLSEELRQIVFRTIESLPEDLRMAITLRELDGLSYEEIAAIMDCPV  178

Consensus       GTVRSRIFRAREAIXXKXXPLJXXAEGKRW                                210
ABF09302, RpoE  GTVRSRIFRAREAIAEKLRPLLGTAEGKRW                                199
BAA10920        GTVRSRIFRAREAIDNKVQPLIRR------                                202
```

Supplementary Figure S3. Alignment of RpoE orthologs from *C. metallidurans* and *E. coli*. RpoE from *C. metallidurans* (ABF09302) and *E. coli* K12 (BBA10920) are also mostly composed of sigma factor domain 2 that binds the -10 region (yellow box), 4.2 binding to the -35 motif (green box) and a linker between both domains.

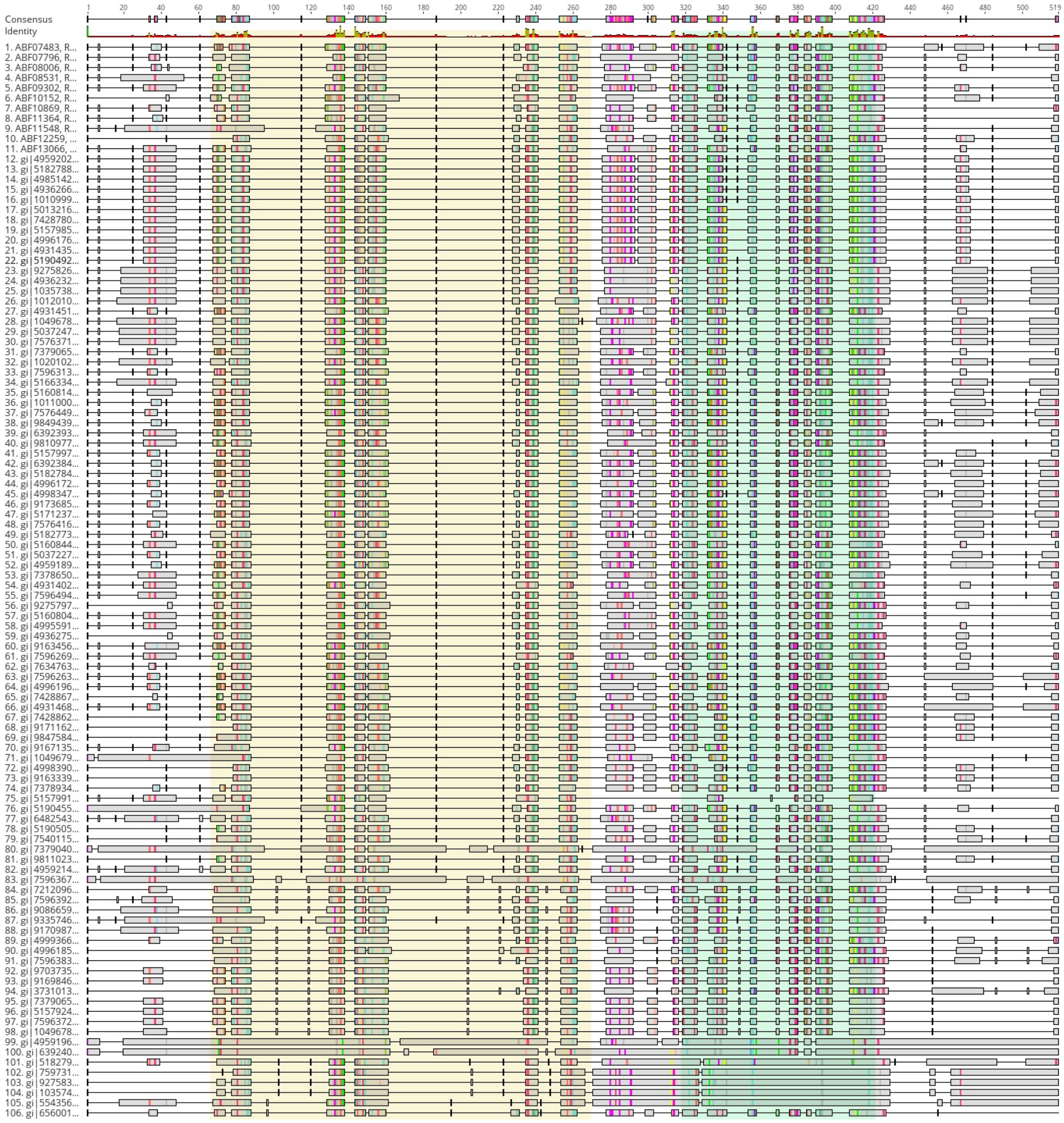

Supplementary Figure S4. Alignment the ECF sigma factors from *C. metallidurans* with 95 other ECF sigma factors.

This alignment of the 11 ECF sigma factors from *C. metallidurans* (on top) with 96 other proteins that were annotated as ECF sigma factors demonstrates that the majority of these sigma factors is mostly composed of sigma factor domain 2 that binds the -10 region (yellow box) and 4.2 binding to the -35 motif (green box). Only a few exceptions exhibited additional amino acid sequences.

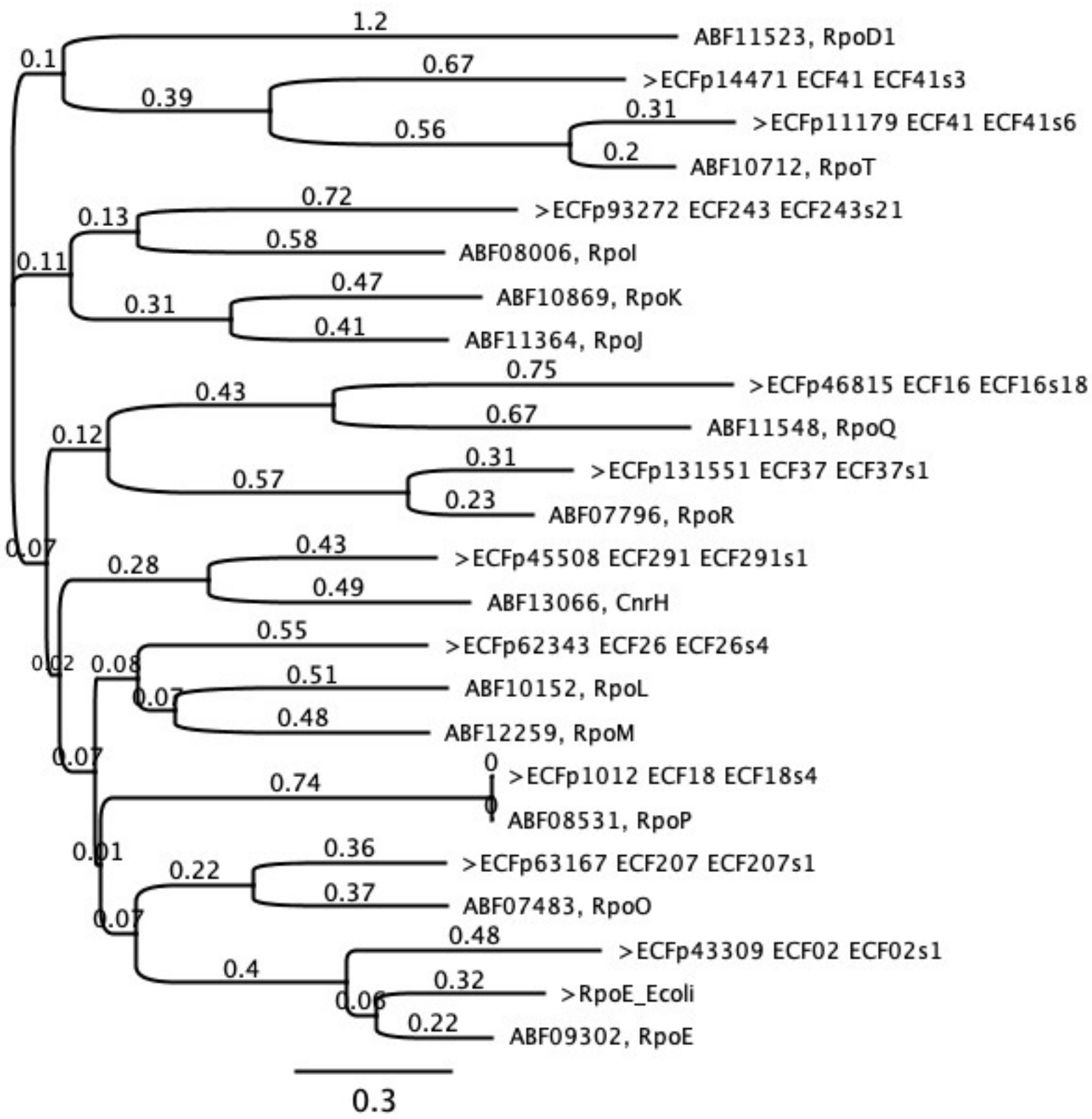

Supplementary Figure S5. Reclassification of the sigma factors from *C. metallidurans*.

BLASTP was used to assign the sigma factors from *C. metallidurans* to representative classes of sigma factors. In the case of ECF sigma factors, these came from the ECF Hub as published (2).

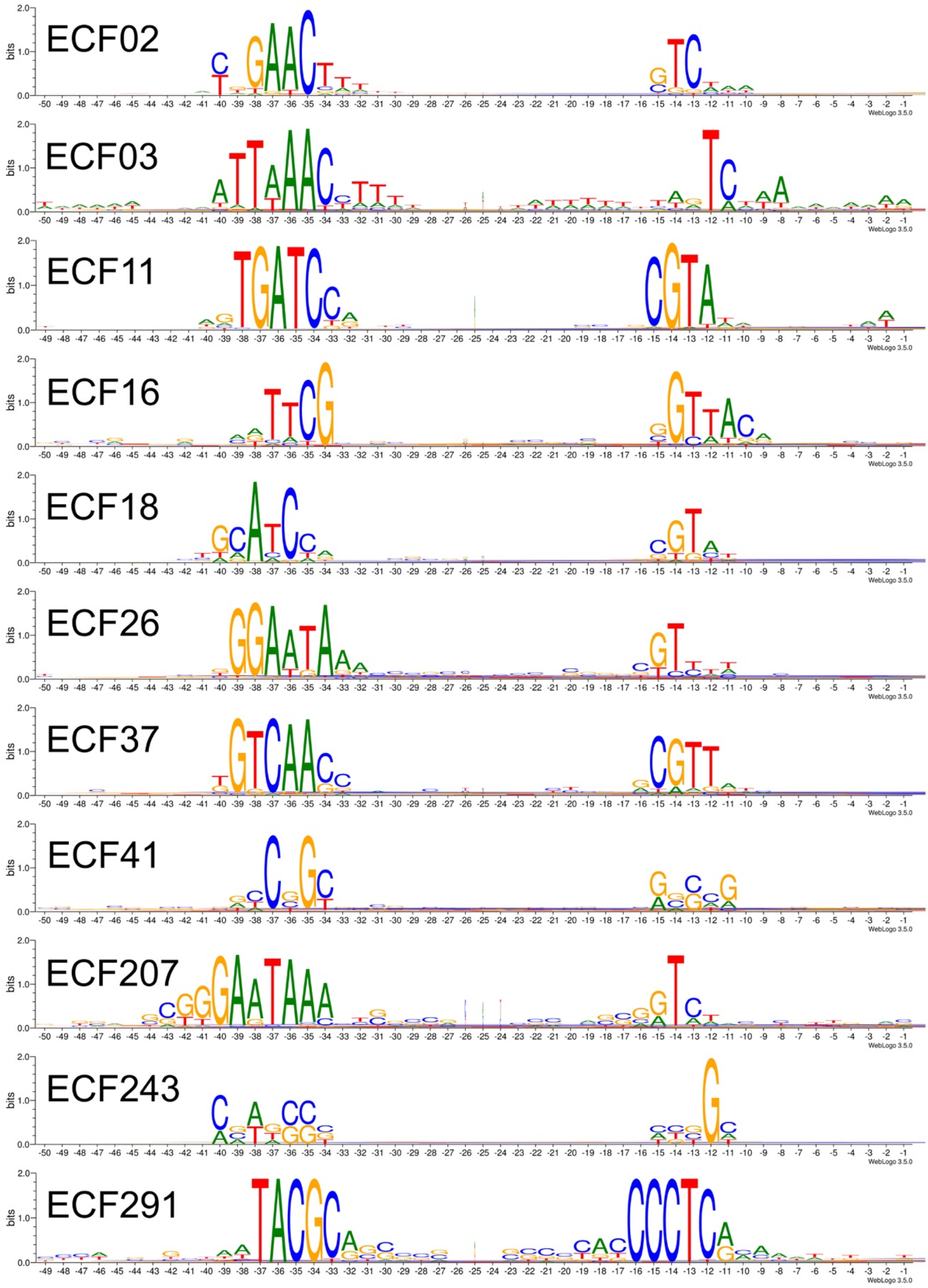

Supplementary Figure S6. Predicted consensus sequences for groups of ECF sigma factors.

The ECF sigma factors from *C. metallidurans* were assigned to ECF groups using ECF hub and the predicted promoter consensus sequences were taken from this data base (2). ECF03 and ECF11 had no representatives in *C. metallidurans*. ECF03 was not further used but ECF11 was used as a control.

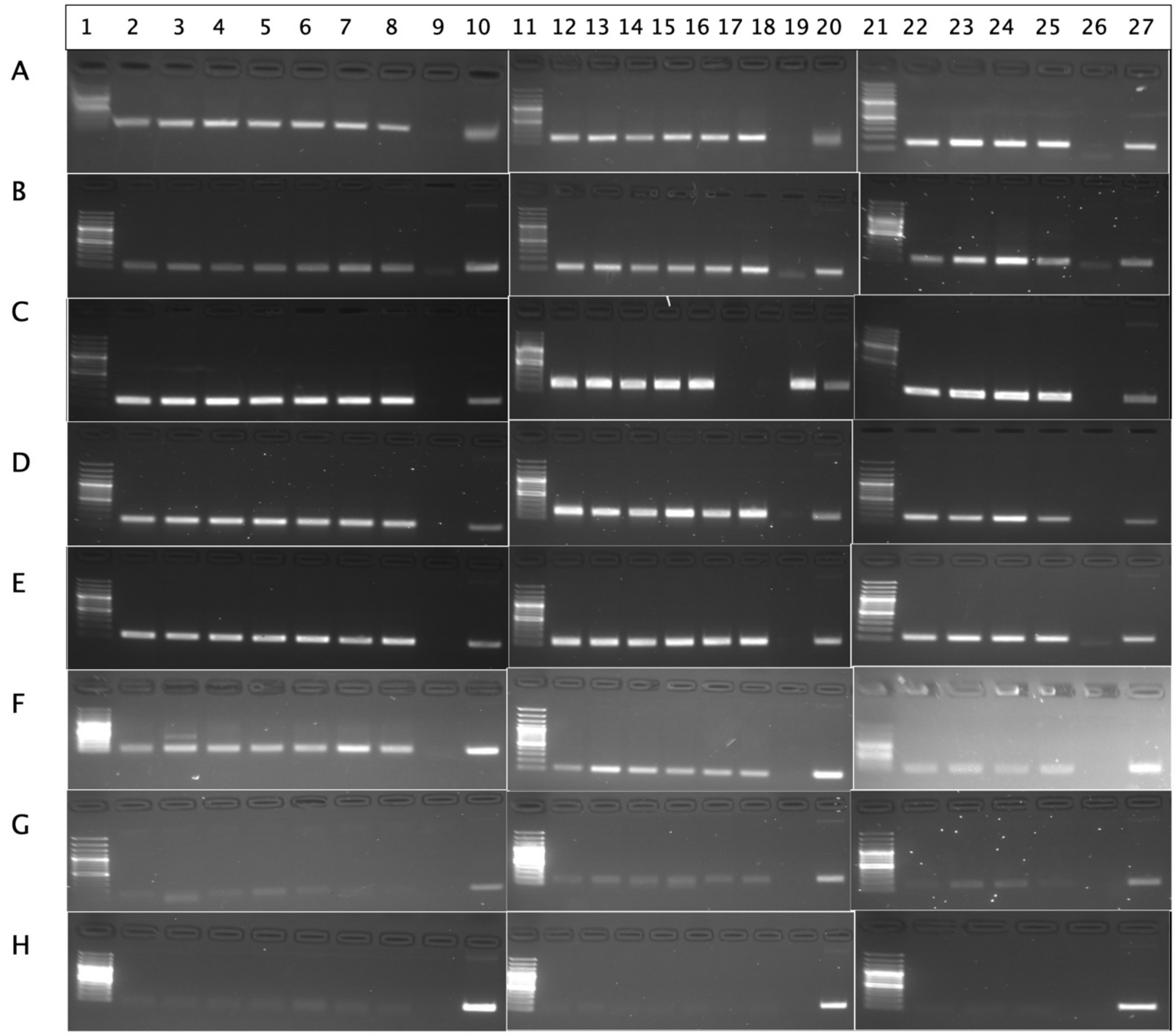

Supplementary Figure S7. Expression of *ssrS* in *C. metallidurans*.

RT-PCR was used to investigate the growth phase-dependent expression of *ssrS* specifying the 6S RNA in *C. metallidurans* strains AE104 and CH34. Panels A (primer *ssrSI*, hexanucleotides), B (*ssrSII*, hexanucleotides), C (*ssrSII*, down1809), D (*ssrSI*, down primer), E (*ssrSII*, down primer), F (*rpoZ* control), G (antisense control) and H (DNA control). Lanes 1, 11, 21 100 bp ladder plus; 9, 19, 26 water control (18 in Panel C); 10, 20, 27 DNA control; 2, 3, 4 AE104 exponential (120 Klett), late exponential (260 Klett) and stationary (270 Klett); 7, 23, 24 AE104 repeat; 13, 14, 25 AE104 second repeat; 22, 5, 6 same with CH34; 8, not, 12 CH34 repeat; 16, 17, 18 (19 in Panel C) second repeat. No evidence for a growth-phase dependent expression of *ssrS* was obtained.

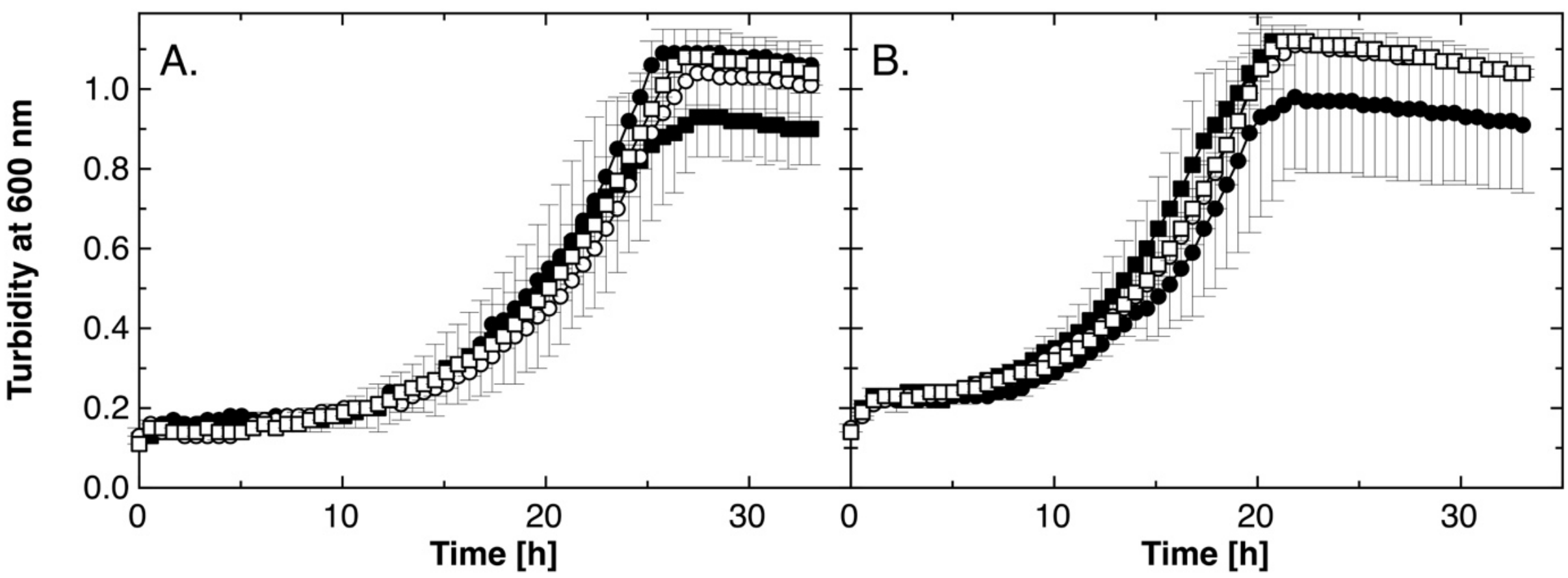

Supplementary Figure S8. Growth of C. metallidurans strains CH34 wild type and AE104 in comparison with their ∆ssrS deletion mutants.

The cells were diluted 50-fold (Panel A) or 20-fold (Panel B) in fresh TMM and incubated with shaking at 30°C. Parents (closed symbols), mutants (open symbols), CH34 (squares) and AE104 (circles)*.* The results of three independent biological replicates are shown and deviations are indicated.

Supplementary Table S1. Scores for the bases in the promoters of sigma factors

Position		-35.6						15.8	-14	-13	-12	-11	-10	-9	-8	-7
RpoD	X	T	T	G	A	C	A		G	X	T	A	T	A	A	T
	0	3	1.5	1.5	1	1	1		0	0	1	2	1	1	1	2
RpoH	C	T	T	G	A	A	A		C	X	T	A	T	T	T	T
	2	2	1	1	1	1	1		2	0	1	1	1	1	1	2
RpoS	G	C	T	G	A	C	A		G	C	T	A	A	A	C	T
	2	2	1	1	1	1	1		1	1	1	1	1	1	1	1
FliA	T	A	A	A	G	T	T		C	C	G	A	T	A	A	T
	2	1	2	1	1	1	1		1	1	1	1	1	1	1	1
ECF02	X	T	T	G	A	A	C		X	X	G	T	C	T	A	A
RpoE	0	1	1	1	2	2	2		0	0	1	2	2	1	1	1
ECF11	X	G	T	G	A	T	C		X	X	C	G	T	A	T	A
	0	0	2	2	2	2	2		0	0	2	2	2	1	0	0
ECF16	X	A	A	T	T	C	G		X	X	G	G	T	T	A	C
RpoQ	0	0	2	1.5	1	1.5	3		0	0	1	2	1.5	1	1.5	1
ECF18	X	G	C	A	T	C	C		X	X	C	G	T	A	T	X
RpoP	0	1	1	3	1	2	1		0	0	1	2	2	1	1	0
ECF26	X	G	G	A	A	T	A		X	X	C	G	T	T	T	T
RpoL,M	0	2	2	2	1	1	2		0	0	1	2	2	1	0	1
ECF37	X	G	T	C	A	A	C		X	X	C	G	T	T	A	T
RpoR	0	2	1	2	2	2	1		0	0	2	2	2	2	1	0
ECF207	X	G	A	A	T	A	A		X	X	G	G	T	C	T	X
RpoO	0	2	2	2	2	2	2		0	0	0	1	3	1	0	0
ECF243	X	A	G	A	G	C	C		X	X	C	C	G	G	C	X
RpoIJK	0	2	1	2	1	2	2		0	0	1	1	1	3	1	0
ECF291	X	A	T	A	C	G	C		X	X	C	C	C	T	C	A
CnrH	0	2	2	2	2	2	1		0	0	2	2	2	2	1	1

Supplementary Table S2. Testing the scores for the promoter motifs against each other

Sigma factor or ECF group	RpoD	RpoH	RpoS	FliA	RpoE ECF02	none ECF11	RpoQ ECF16	RpoP ECF18	RpoL, M ECF26	RpoR ECF37	RpoO ECF207	RpoI, J, K ECF243	CnrH ECF291
RpoD	17	11	9	5	6	10	4.5	5	5	3	5	2	2
RpoH	14	18	5	4	8	8	2.5	3	6	6	7	0	4
RpoS	12	8	15	4	4	7	1.5	3	3	0	2	3	3
FliA	7	6	2	17	2	5	6	6	6	3	7	5	4
ECF02	8	7	3	2	17	9	3.5	1	1	6	2	2	8
ECF11	6	5	4	3	7	18	3.5	9	8	9	6	3	6
ECF16	3	2	1	6	3	5	17	7	5	7	8	4	4
ECF18	3	2	2	3	2	10	6	15	9	9	10	7	5
ECF26	5	7	2	4	3	11	4.5	10	16	10	10	3	6
ECF37	6.5	7	2	3	9	13	6	7	9	16	8	3	7
ECF207	2	4	1	5	3	5	7.5	10	10	8	17	2	2
ECF243	1	0	4	2	2	5	1.5	7	5	3	2	14	8
ECF291	1.5	2	2	2	7	7	1	5	4	5	2	9	21
Rmet_0598p@RpoO	3	6	5	8	3	5	7.5	11	10	8	16	2	2
TSS_640976+2	9	7	10	4	4	8	1.5	3	3	5	3	6	8
Rmet_1649@RpoP	3	2	2	5	3	9	6	15	9	9	10	7	6
TSS_1783261+2	11.5	9	4	5	9	9	1.5	4	3	7	0	3	7
Rmet_3281@RpoL	4	6	4	3	4	8	4.5	9	14	8	10	2	5
TSS_3556242-2	12	9	6	3	7	9	4.5	3	4	5	3	1	5
TSS_3556433-2	8	5	8	2	2	6	4.5	3	2	3	0	8	7
TSS_3556965-2	5.5	4	8	2	5	3	3	1	2	2	2	2	5

The consensus sequences of sigma factors were scored with that of all the other sigma factors in *C. metallidurans*, except RpoN, which was not considere ECF11 served as a control since this group has no representative in *C. metallidurans*. Yellow fields indicate cross-scoring of sigma factors in the case of RpoD and RpoS as one group and ECF11, 18, 26, 37 and 207 as another group. In the box, the Rmet open reading frames with “p” for promoter indicate predicted pro for the indicated sigma factors (7), however, no TSSs could be found at the corresponding position. The measured TSSs for the respective genes are indicated

Supplementary Table S3. Table Overview Promoters including the second-best consensus motifs for sba and ba promoters[a]

Gene(s)	Promoter (italics not regulated)	Second-best -10 or -35 site for sba respectively ba prom.
IM uptake		
zupT	PosFail (RpoD); RpoD	
pitA	RpoDS; *RpoD*	
corA1	sba(RpoS);	S-10@RpoS#0.89/0.75
corA2	*PosFail (RpoS);*	
corA3	*sba(RpoD);*	*S-10@ECF207#0.60/0.88*
zntB	sba(RpoD);	S-10@FliA#0.50/0.63
hoxN	*sba(RpoD); sba(RpoD); sba(RpoD);*	*S-10@RpoHRpoS#0.44/0.75;S-10@RpoH#0.44/0.75;* S-10@FliA#0.50/0.88
feo	RpoD	
mgtA	*RpoS;*	
mgtB	PosFail (ECF291); ba(RpoDS);	S-35@ECF291#0.45/0.50
pstSCABU	sba(RpoD);	S-10@RpoH#0.67/0.75
IM Efflux		
zntA	RpoD	
cadA	sba(RpoD); RpoDS; RpoD	S-10@FliA#0.50/0.75
pbrA	RpoD	
czcP	sba(RpoDS); RpoD	S-10@ECF291#0.50/0.63
cupA	sba(RpoD);	S-10@RpoHRpoS#0.44/0.63
copF	sba(RpoD); PosFail (RpoD);	S-10@ECF207#0.60/0.75
ctpA1	sba(ECF291);	S-10@RpoH#0.67/0.75
rdxI	sba(RpoD);	S-10@RpoH#0.78/0.75
czcD	sba(RpoD); PosFail(RpoDS); sba(RpoS);	S-10@RpoH#0.67/0.75; S-10@RpoS#0.67/0.50
dmeF	sba(RpoD); sba(RpoD);	S-10@ECF207#0.60/0.75; S-10@RpoS#0.78/1.00
fieF	RpoH; sba(RpoD);	S-10@RpoH#0.67/0.75
cdfX	RpoD	
chr1	RpoDS	
chr2	RpoD; RpoD; sba(RpoD);	S-10@ECF16#0.56/0.50
ars	sba(RpoD);	S-10@FliA#0.50/0.75
atmA	RpoDS; sba(RpoD);	S-10@RpoH#0.78/0.75
Phosphate et al.		
ppx	*sba(RpoD);*	S-10@ECF11#0.71/0.75
ppk	sba(RpoDS); sba(RpoDS);	S-10@RpoS#0.44/0.63; S-10@RpoS#0.78/0.75
gshAB	RpoD; sba(RpoDRpoH); sba(RpoD);	S-10@RpoH#0.78/0.75; S-10@RpoS#0.67/0.88
bfd, bfr	RpoD	
grxC	sba(RpoD);	S-10@FliA#0.63/1.00
Isc	sba(RpoD); sba(RpoD); sba(RpoDS);	S-10@ECF207#0.80/0.75; S-10@RpoHRpoS#0.44/0.75; S-10@RpoS#0.56/0.75

zur, cobW2	sba(RpoDS); *sba(RpoD);*	S-10@RpoS#0.89/0.75; S-10@FliA#0.88/0.75
cobW3	sba(RpoD); sba(RpoDS);	S-10@ECF243#0.43/0.63; S-10@RpoS# .56/0.75
cobW1 cluster	RpoDS;	
Regulators		
furA	*sba(RpoD);*sba(RpoD)	S-10@RpoH#0.78/0.88; S-10@RpoH#0.56/0.63
furB	*RpoDS;* sba(RpoD)	S-10@FliA#0.50/0.63
phoB	sba(RpoD);	S-10@FliA#0.50/0.75
phoR	RpoS	
phoU	*sba(RpoD); RpoD*	*S-10@RpoH#0.44/0.75*
iscR, isc	RpoD	
dksA	sba(RpoS); sba(ECF26ECF207); PosFail (RpoS);	S-10@ECF243#0.71/0.50; S-10@ECF207#0.80/0.63
zur	sba(RpoDS); sba(RpoD)	S-10@RpoS#0.89/0.75; S-10@FliA#0.88/0.75
zntR	sba(RpoDS);	S-10@RpoS#0.89/0.75
cadR	RpoDS; PosFail (FliAECF243)	
pbrR	RpoD;	
cupR	sba(RpoD)	S-10@RpoHRpoS#0.44/0.63
arsR	sba(RpoD);	S-10@FliA#0.50/0.75
czcRS	sba(RpoD); sba(RpoD); sba(RpoD);	S-10@RpoS#0.56/0.63; S-10@RpoHRpoS#0.44/0.63; S-10@ECF291#0.40/0.63
czcR2S2	*sba(RpoD);* PosFail (RpoD); RpoD;	*S-10@FliA#0.50/0.75*
agrRS	RpoDS; RpoD; *sba(RpoD)*;sba(RpoD); sba(RpoD)	*S-10@RpoH#0.44/0.75;* S-10@ECF207#0.80/0.88; S-10@ECF243#0.71/0.63
copR1S1	*RpoD;* sba(RpoD); sba(RpoS); sba(ECF16); sba(RpoDS)	S-10@RpoH#0.56/0.75; S-10@RpoS#0.56/0.75; S-10@FliA#0.75/0.63; S-10@RpoH#0.67/0.75
copR2S2	*sba(RpoD);* sba(RpoD); sba(RpoD);	S-10@FliA#0.50/0.75; S-10@RpoH#0.56/0.63; S-10@RpoH#0.67/0.75
zniR	sba(RpoD)	S-10@RpoH#0.67/0.88
zniS	sba(RpoD); sba(RpoD); RpoD; sba(RpoDRpoH); sba(RpoD); *sba(RpoD);*	S-10@RpoH#0.67/0.75; S-10@FliA#0.63/0.75; S-10@RpoH#0.67/0.75; S-10@FliAECF02#0.50/0.63; S-10@RpoH#0.56/0.63
zneP,zneR,zneS	PosFail (ECF291);	
zneR2S2	*sba(RpoDRpoH);*	*S-10@RpoH#0.67/0.75*
hmzR	RpoD;	
hmzS	sba(RpoS);	S-10@RpoS#0.44/0.50
Periplasmic Functions		
czcN	sba(RpoD);	S-10@RpoHRpoS#0.56/0.75
Rmet_6368, czcICBA	sba(RpoD);	S-10@RpoH#0.67/0.88
czcICBA	*sba(ECF16);* sba(RpoDS); sba(RpoDS); sba(RpoDS);	S-10@ECF243#0.57/0.25;S-10@FliA#0.88/0.88; S-10@FliA#0.88/0.88; S-10@FliA#0.88/0.88
czcA	sba(RpoD); sba(ECF11); sba(RpoD);	S-10@ECF243#0.57/0.63; S-10@ECF207#0.60/0.75; S-10@RpoH#0.67/0.75
czcE	sba(RpoD); RpoD; sba(RpoD);	S-10@RpoHRpoS#0.44/0.75;

		S-10@RpoHRpoS#0.44/0.75
czcJ	sba(RpoD); sba(RpoD); sba(RpoD); sba(RpoDS);	S-10@ECF207#0.60/0.75; S-10@FliA#0.75/0.75;<br>S-10@RpoS#0.89/0.75;S-35@RpoHECF243#0.43/0.61
czcI2C2B2'	RpoD	
czcB2''A2	sba(RpoH);	S-10@RpoH#0.56/0.63
ncc	sba(RpoSECF243); RpoDS; PosFail (RpoDS)	S-10@ECF243#0.71/0.50
zneBA	sba(RpoD);	S-10@RpoH#0.44/0.75
zneC	sba(RpoD);	S-10@ECF207#0.80/0.75
hmvBA'	PosFail (RpoD);	
hmvA''	RpoD;	
hmyFCB	sba(RpoH); PosFail (RpoD); sba(RpoD);	S-10@RpoH#0.78/0.75; S-10@ECF207#0.80/0.75
hmyA	sba(RpoD);	S-10@RpoHRpoS#0.44/0.63
nimBA2	sba(RpoDS);	S-10@RpoS#0.78/0.88
nimA1C	*sba(RpoD);*	*S-10@FliA#0.63/1.00*
hmzBA	sba(RpoD); sba(RpoD);	S-10@RpoS#0.44/0.63; S-10@FliA#0.50/0.63
cusDCBA	RpoD;	
cusF	RpoDS;	
silDCBA	PosFail (ECF37); *RpoD;* sba(RpoD)	S-10@FliA#0.63/0.75
copQ	sba(RpoH);	S-10@RpoH#0.67/0.63
copOFGJ	sba(RpoDS); sba(RpoDS);	S-10@RpoS#0.67/0.75; S-10@RpoS#0.67/0.75
copN	sba(RpoDS); sba(ECF37);	S-10@ECF207#0.60/0.75; S-10@FliA#0.63/0.75
copTV	ba(RpoD); sba(ECF291); sba(RpoD); sba(RpoD);	S-35@RpoH#0.71/0.67; S-10@ECF243#0.57/0.63;<br>S-10@ECF243#0.57/0.75;S-10@RpoHRpoS#0.44/0.75
copM	PosFail (ECF02);	
copK	RpoDS; sba(RpoD); RpoD	S-10@ECF243#0.71/0.63
copA1B1C1D1I	*sba(RpoD);* sba(RpoD); RpoH; ECF02	*S-10@RpoH#0.56/0.75;* S-10@FliA#0.50/0.75
copL	sba(RpoD);	S-10@RpoS#0.56/0.75
copEW	ba(RpoDECF11); sba(RpoD);	S-35@ECF11#1.00/0.78; S-10@ECF207#0.60/0.75
$copA_2B_2C_2D_2$	ECF16;	
cnrYXHCBA	sba(RpoD); sba(RpoD); sba(RpoD); sba(RpoDS);<br>sba(RpoD);	S-10@FliAECF02#0.50/0.63; S-10@RpoS#0.44/0.63;<br>S-10@ECF207#0.60/0.63;S-10@ECF207#0.80/0.88;<br>S-10@FliA#0.63/0.75
cnrA	sba(ECF207);	S-10@FliA#0.88/0.75
cnrT	RpoD; sba(RpoD); sba(RpoD); sba(RpoD); *sba(RpoD);*	S-10@FliA#0.50/0.75; S-10@ECF207#0.80/0.75;<br>S-10@RpoH#0.56/0.75;*S-10@RpoHRpoS#0.44/0.75*
Sigma factors		
dnaG, rpoD1	*sba(RpoDS);*	S-10@RpoS#0.67/0.88
rpoD2	sba(RpoD); *RpoD*	S-10@RpoH#0.67/0.75
rpoS	sba(RpoD); *RpoD*; RpoD	S-10@RpoS#0.56/0.63
rpoH	PosFail (RpoDS); sba(RpoDS);	S-10@RpoS#0.78/0.75

fliA (Op1026f_3)	*sba(ECF16);* sba(ECF207); sba(RpoD); RpoD; sba(RpoD); ba(ECF02); *sba(RpoD);*RpoD;	*S-10@FliA#0.50/0.75*; S-10@ECF02#0.63/0.13; S-10@FliA#0.50/0.75; S-10@RpoH#0.44/0.63; S-35@RpoH#0.71/0.89; *S-10@FliA#0.50/0.75*
acrD,rpoI,rsiA, Op0320r_2	RpoD;	
rpoJ, rsjA	sba(RpoD);	S-10@RpoH#0.56/0.75
Rmet_4002, rpoK, AST, rskA, Op1123r_1	*sba(RpoD);*	*S-10@RpoH#0.67/0.88*
Rmet_3281, rpoL, rslA, yedYZ;	*PosFail (RpoDS);*	
rpoM, rsmA, Op1608f	*sba(RpoD);*	*S-10@RpoH#0.67/0.88*
3 ASTs, rpoO, rsoA (2427 bp to rpoO)	sba(RpoDS);	S-10@RpoS#0.67/0.75
rpoE, rseA, rseB	*sba(RpoD); RpoD*	S-10@RpoS#0.56/0.88
rseA, rseB, Rmet_6525	sba(RpoDS); sba(ECF291);	S-10@RpoS#0.56/0.50; S-10@ECF16#0.56/0.25
cnrYXHCB	sba(RpoD); sba(RpoD);	S-10@RpoS#0.44/0.63; S-10@FliA#0.63/0.75
creD, rpoP, rspA rpoQ, rsqA, Op1356f_1	sba(RpoS); PosFail (RpoDS); *sba(RpoD);* PosFail (ECF02);	S-10@RpoS#0.44/0.63; S-10@RpoS#0.78/0.63
2 ASTs, gigP, AST(rpoQ), gigA, gigB,gigT	RpoH;	
rpoR, rsrA, rsrB	RpoD;	

The predicted promoters upstream of the TSSs for the respective genes or operons were transferred from the Supplementary Material (Landscape.xlxs) as Regions.xlsx (Supplement). From here, only the promoter associations were used. *Italics* indicate a TSS that was not regulated under changing metal availability *(Reg_2 and NONE).* Grey fields indicate a possible contribution made by an alternative sigma factor. For a sba promoter, the -10 sequence (S-10) was separately associated with the consensus sequences of the sigma factors, except for RpoD. The best-scoring sigma factor is indicated. After the hash-tag (#) the ratio of the relative score of this sigma factor (score for this specific sequence divided by the score to its consensus sequence) in comparison (/) of the relative score for RpoD is shown. For the ba-promoters, the same was done for the -35 sequences. These assignments were in bold or italics, similar to their promoters to increase readability. PosFail promoters and clearly assigned promoters were not considered.

Supplementary Table S4. Transcriptional landscape of the cnr resistance determinant on plasmid pMOL28

Element	locus tag	start	stop	gene	Max	Reg	Operon/Sigma	Impact
**TSS_52310+5**	Rmet_6205:999	52310			119	REG_5@CM	sba(RpoD); (11/6);	cnrY;cnrX;cnrH;cnrC;cnrB;
**TSS_52343+5**	Rmet_6205:966	52343			1,227	REG_3@CM	sba(RpoD); (12/7); Pub((m)-sba)	cnrY;cnrX;cnrH;cnrC;cnrB;
TSS_52420+5	*Rmet_6205:889*	*52420*			*25*	*REG_5@CM*	*sba(RpoD); (12/3);*	*cnrY;cnrX;cnrH;cnrC;cnrB;*
**TSS_53103+5**	Rmet_6205:206	53103			82	REG_16@C0	sba(RpoDS); (14/9); Pub();	cnrY;cnrX;cnrH;cnrC;cnrB;
**TSS_53286+5**	Rmet_6205:23	53286		*cnrYp*	28,233	REG_11@CM	sba(RpoD); (9/-1); Pub(s)	cnrY;cnrX;cnrH;cnrC;cnrB;
NPKM_6205	Rmet_6205	53309	53597	*cnrY*	1081	REG_7@CM	Op1896f_2	P56621 Nickel and cobalt resistance protein cnrY
NPKM_6206	Rmet_6206	53593	54040	*cnrX*	726	REG_8@CM	Op1896f_2	P37975 Nickel and cobalt resistance protein cnrR
NPKM_6207	Rmet_6207	54036	54612	*cnrH*	466	REG_8@CM	Op1896f_2	P37978 RNA polymerase sigma factor cnrH
NPKM_6208	Rmet_6208	54684	55941	*cnrC*	255	REG_5@CM	Op1896f_2	P37974 Nickel and cobalt resistance protein cnrC
NPKM_6209	Rmet_6209	55937	57125	*cnrB*	150	REG_6@CM	Op1896f_2	P37973 Nickel and cobalt resistance protein cnrB
TSS_55986+5	*Rmet_6210:1135*	*55986*			*36*	*REG_7@CM*	*sba(ECF207); (11/5);*	*cnrA;*
NPKM_6210	Rmet_6210	57121	60352	*cnrA*	168	REG_6@CM	Op1896f_2	P37972 Nickel and cobalt resistance protein cnrA
TSS_57551+5	*Rmet_6211:2846*	*57551*			*28*	*REG_6@CM*	*not assigned*	*cnrT;*
**TSS_57815+5**	Rmet_6211:2582	57815			136	REG_27@CE	RpoD; (11/11); Pub(m)	cnrT;
**TSS_57980+5**	Rmet_6211:2417	57980			114	REG_3@CM	sba(RpoD); (10/-3); Pub()	cnrT;
TSS_59089+5	*Rmet_6211:1308*	*59089*			*42*	*REG_8@CM*	*sba(RpoD); (9/-6);*	*cnrT;*
**TSS_59447+5**	Rmet_6211:950	59447			87	REG_17@CM	sba(RpoD); (12/-2);	cnrT;
TSS_59634+5	Rmet_6211:763	59634			870	NONE	sba(RpoD); (12/3); Pub(no)	cnrT;
NPKM_6211	Rmet_6211	60397	61453	*cnrT*	230	REG_5@CM	Op1896f_2	Q9L3G0 CnrT protein
3UTR_6211	Rmet_6211	61454	63472	*cnrT*	29	REG_2@CM	Op1896f_2	Q9L3G0 CnrT protein

[a]Element is the name of the TSS, 5UTR, 3UTR, or gene; "NPKM" indicates gene regions and corresponds to the Rmet-numbering. Locus tag is either the Rmet name, or in the case of TSSs, the closest gene:distance to it in base pairs. The TSSs were annotated to a gene when upstream of it, but were annotated to an AST when within the AST. Nevertheless, the distance to the next open reading frame was always indicated. Regulated TSSs are in bold, those with a score below the cut-off of 50 are in italics. Start and stop indicate the positions of the elements. Gene is the annotated gene of an NPKM gene region, 3UTR or 5UTR. Max gives the highest TSS score under all conditions or the highest NPKM values of the other elements. Reg shows if an element is regulated (REG), or not (NONE), the -fold ratio between highest and lowest level, and the conditions under which the respective highest value was observed. Operon/Sigma defines either the predicted operon of the sense elements or the prediction of the sigma factor associated with a TSS. The impact of a sense element is the function or the predicted gene product of the gene or the UTRs associated with it. Impact of an antisense element represents the corresponding sense elements with which the AST is interacting. Impact of a TSS indicates the sense or antisense elements influenced by this TSS. The ASTs of the *cnr* region are provided in the Supplement. The *cnr* TSSs have been previously published (8) but were further analyzed here with respect to the sigma factor responsible for the respective transcription initiation event.

Supplementary Table S5. Bacterial strains and plasmids used

Strain	Description	Reference
CH34	Wild type	(9)
AE104	Plasmid-free	(9)
<u>mutants</u>		
DNA78	AE104 Δ*ssrS*	this publication
DNA79	CH34 Δ*ssrS*	this publication
DN658	AE104 Δ*rpoQ*	(10)
<u>plasmids</u>		
VS276	pVDZ'2(*lacZ*)	(11)
ECB297	pVDZ'2(*rpoQp-lacZ*)	This publication
ECB298	pVDZ'2(*rpoRp-lacZ*)	This publication
ECB299	pVDZ'2($rpoO_Np$-*lacZ*)	This publication
ECB301	pVDZ'2(*rpoJp-lacZ*)	This publication
ECB303	pVDZ'2(*rpoEp-lacZ*)	This publication
ECB304	pVDZ'2(*rpoKp-lacZ*)	This publication
ECB305	pVDZ'2(*rpoLp-lacZ*)	This publication
ECB306	pVDZ'2($rpoO_Wp$-*lacZ*)	This publication
ECB307	pVDZ'2(*rpoPp-lacZ*)	This publication
ECB308	pVDZ'2($rpoE_2p$-*lacZ*)	This publication
ECB311	pVDZ'2(*rpoIp-lacZ*)	This publication
ECB312	pVDZ'2(*rpoMp-lacZ*)	This publication
ECB314	pVDZ'2($copA_2p$-*lacZ*)	This publication

References of the Supplement

1. Zhang Y, Feng Y, Chatterjee S, Tuske S, Ho MX, Arnold E, Ebright RH. 2012. Structural Basis of Transcription Initiation. Science 338:1076–1080.
2. Casas-Pastor D, Muller RR, Jaenicke S, Brinkrolf K, Becker A, Buttner MJ, Gross CA, Mascher T, Goesmann A, Fritz G. 2021. Expansion and re-classification of the extracytoplasmic function (ECF) sigma factor family. Nucleic Acids Res 49:986–1005.
3. Helmann JD. 2002. The extracytoplasmic function (ECF) sigma factors. Adv Microb Phys 46:47–110.
4. Kitagawa M, Wada C, Yoshioka S, Yura T. 1991. Expression of ClpB, an analog of the ATP-dependent protease regulatory subunit in *Escherichia coli*, Is controlled by a heat-shock sigma factor (Sigma-32). J Bacteriol 173:4247–4253.
5. Bouillet S, Bauer TS, Gottesman S. 2024. RpoS and the bacterial general stress response. Microbiol Mol Biol Rev 88:e0015122.
6. Große C, Grau J, Große I, Nies DH. 2022. Importance of RpoD- and non-RpoD-dependent expression of horizontally acquired genes in *Cupriavidus metallidurans*. Microbiol Spectr 10:e00121–22.
7. Todor H, Osadnik H, Campbell EA, Myers KS, Li H, Donohue TJ, Gross CA. 2020. Rewiring the specificity of extracytoplasmic function sigma factors. Proc Natl Acad Sci U S A 117:33496–33506.
8. Große C, Grau J, Herzberg M, Nies DH. 2024. Antisense transcription is associated with expression of metal resistance determinants in *Cupriavidus metallidurans* CH34. Metallomics 16:mfae057.
9. Mergeay M, Nies D, Schlegel HG, Gerits J, Charles P, van Gijsegem F. 1985. *Alcaligenes eutrophus* CH34 is a facultative chemolithotroph with plasmid-bound resistance to heavy metals. J Bacteriol 162:328–334.
10. Große C, Poehlein A, Blank K, Schwarzenberger C, Schleuder G, Herzberg M, Nies DH. 2019. The third pillar of metal homeostasis in *Cupriavidus metallidurans* CH34: Preferences are controlled by extracytoplasmic functions sigma factors. Metallomics 11:291–316.
11. Große C, Anton A, Hoffmann T, Franke S, Schleuder G, Nies DH. 2004. Identification of a regulatory pathway that controls the heavy metal resistance system Czc via promoter *czcNp* in *Ralstonia metallidurans*. Arch Microbiol 182:109–118.
